# Targeting a Novel G-Quadruplex in the *CARD11* Oncogene Promoter with Naptho(2,1-b)furan-1-ethanol,2-nitro- Requires the Nitro Group

**DOI:** 10.3390/genes13071144

**Published:** 2022-06-25

**Authors:** Kennith Swafford, Baku Acharya, Ying-Zhi Xu, Thomas Raney, Mason McCrury, Debasmita Saha, Brendan Frett, Samantha Kendrick

**Affiliations:** 1 Department of Biochemistry and Molecular Biology, University of Arkansas for Medical Sciences, Little Rock, AR 72205, USA; krswafford@uams.edu (K.S.); yxu2@uams.edu (Y.-Z.X.); raneytd@hendrix.edu (T.R.); mjmccrury@uams.edu (M.M.); 2 Department of Pharmaceutical Sciences, University of Arkansas for Medical Sciences, Little Rock, AR 72205, USA; bacharya@uams.edu (B.A.); dsaha@uams.edu (D.S.)

**Keywords:** G-quadruplex, diffuse large B cell lymphoma, transcription analogs

## Abstract

The aggressive nature of the activated B cell such as (ABC) subtype of diffuse large B cell (DLBCL) is frequently associated with altered B cell Receptor (BCR) signaling through the activation of key components including the scaffolding protein, *CARD11*. Most inhibitors, such as ibrutinib, target downstream BCR kinases with often modest and temporary responses for DLBCL patients. Here, we pursue an alternative strategy to target the BCR pathway by leveraging a novel DNA secondary structure to repress transcription. We discovered that a highly guanine (G)-rich element within the *CARD11* promoter forms a stable G-quadruplex (G4) using circular dichroism and polymerase stop biophysical techniques. We then identified a small molecule, naptho(2,1-b)furan-1-ethanol,2-nitro- (NSC373981), from a fluorescence-resonance energy transfer-based screen that stabilized *CARD11* G4 and inhibited *CARD11* transcription in DLBCL cells. In generating and testing analogs of NSC373981, we determined that the nitro group is likely essential for the downregulation of *CARD11* and interaction with *CARD11* G4, and the removal of the ethanol side chain enhanced this activity. Of note, the expression of *BCL2* and *MYC*, two other key oncogenes in DLBCL pathology with known promoter G4 structures, were often concurrently repressed with NSC373981 and the highly potent **R158** analog. Our findings highlight a novel approach to treat aggressive DLBCL by silencing *CARD11* gene expression that warrants further investigation.

## 1. Introduction

Diffuse large B cell lymphoma (DLBCL) is the most prevalent non-Hodgkin’s lymphoma with the activated B cell (ABC) subtype accounting for at least a third of the cases. Despite the successful disease-free survival achieved in about 60% of DLBCL, there is no effective long-term treatment for the majority of patients with ABC DLBCL and for a subset of patients with the more favorable germinal center B cell (GCB) cell-of-origin DLBCL subtype [1,2,3,4,5]. Unlike other DLBCL subtypes, the malignant transformation of ABC DLBCL frequently involves aberrant B cell receptor (BCR) activation through concurrent or independent mutations of the two signaling subunits of BCR, CD79A/B (24% of cases), or downstream molecules, CARD11 (10%) and MYD88 (35%), providing tumor cells with a constitutive activation of these survival and proliferation pathways [6,7,8,9]. Even beyond activating mutations, there is a recognized oncogenic-dependent survival of ABC DLBCL on the expression of BCR molecules. Studies using RNA interference to inhibit BCR signaling in ABC DLBCL demonstrate a significant decrease in cell viability, indicating an addictive reliance on the expression of BCR-related proteins [6]. Furthermore, newly identified molecular subsets of DLBCL recognize tumor types extending beyond the initial cell-of-origin subgroups with specific dependence on BCR deregulation that correlate to poor prognosis [10,11,12]. As such, the clinical importance of BCR signaling inhibition is well appreciated and thus far led to the development of kinase or protease inhibitors. Although ibrutinib (Bruton’s Tyrosine Kinase (BTK) inhibitor) shows promise in chronic myeloid and lymphocytic leukemia patients, this therapeutic approach fails to achieve complete, sustained responses in DLBCL patients because of inherent resistances due to the additional genetic lesions in other components of the BCR pathway and acquired mutations in BTK [8,13,14]. However, a recent phase III clinical trial testing the efficacy of ibrutinib in combination with the standard R-CHOP chemotherapy indicates a potential three-year survival benefit for a subset of younger DLBCL patients [15]. In addition to BTK, other kinases in the pathway are targeted with small molecules including, fostamatinib (SYK inhibitor) and dasatinib (LYN inhibitor) but also tend to demonstrate greater response rates in leukemias [13,16]. Yet another approach is to block BCR signaling further downstream with a MALT1 protease inhibitor, MI-2, as previously explored in ABC cell line and mouse models, but whether induced mutations within the MALT1 protease active site itself will lead to resistance in patients remains unknown [17]. Identifying new strategies to target this oncogenic signaling will advance the knowledge of DLBCL pathobiology and the treatment of patients with this refractory-prone disease.

We propose that targeting G-quadruplex (G4) DNA secondary structures within promoter regions of BCR-related signaling molecules will restrict gene production and subsequent translation into protein to potentially minimize the risk of resistance. G-rich sequences consisting of at least four runs of two or more Gs can adopt a G4 structure that consists of stacked G-tetrads stabilized by a monovalent cation such as Na^+^ or K^+^ within DNA during nuclear processes such as transcription because of torsional stress [18,19]. DNA secondary structures are proposed to act as molecular switches, turning gene expression “on” or “off”. In support of this, genome-wide analyses detected DNA secondary structure-forming sequences within close proximity of transcriptional start sites, which were mostly oncogene promoters [20] and we, along with others, previously demonstrated small molecules that stabilize the *MYC* oncogene G4 inhibit promoter activity and subsequent expression [21,22,23,24,25]. In more recent years, the recognition of the utility of G4s to serve as therapeutic targets, particularly against cancer, has extended beyond *MYC* with the continued research into other oncogenes [26,27]. The folding pattern of G4 structures can vary depending on the primary nucleic acid sequence within a given genomic locus. Similarly to other *cis*-regulatory elements, G4s exhibit conserved features essential to basic structure and overall function while maintaining sufficient complexity for selective targeting [26,28]. G4 structures consist of shared core sequences that give rise to the G-tetrads for structure assembly. As a source of diversity, the length and specific nucleotide sequence of the central and lateral loop regions as well as the number of stacked G-tetrads characterize a specific folding pattern and offers unique as well as conserved scaffolds for the binding of small molecules and nuclear proteins [26,27,29,30,31,32].

Based on the importance of CARD11 acting as an anchor molecule to propagate constitutive BCR signaling that promotes the survival of ABC DLBCL [6], we examined the *CARD11* promoter for a potential G4 forming sequence. CARD11 coordinates the formation of a multi-protein complex that counteracts inhibitory molecules of a prosurvival transcription factor, NFkB, and maintains active NFkB to enhance DLBCL growth and survival [33,34,35]. In addition, CARD11 is downstream of the previously sought after BCR related targets and further supports the potential for this molecule to serve as a potent, effective target for BCR-dependent DLBCL. In this study, we discovered the *CARD11* promoter region contains a G-rich sequence with the ability to form a stable G4 structure. We explored its potential as a target for downregulating *CARD11* expression within DLBCL and used a functional group analog approach to develop structure activity relationships with the scaffold. 

## 2. Materials and Methods

### 2.1. Oligonucleotides and G4 Forming Conditions

All oligonucleotide sequences were synthesized by Integrated DNA Technology (IDT; Coralville, IA, USA) or Eurofins (Louisville, KY, USA). Oligonucleotide probes for FRET and DNA polymerase stop assays were purified using a high-performance liquid chromatograph (HPLC), and oligonucleotides for circular dichroism (CD) analysis were salt purified. Oligonucleotide sequences used in this study are listed in Supplementary Table S1. Prior to analysis, all oligonucleotide probes and oligonucleotides were slow-cool annealed to permit G4 formation by heating for 5 min at 95 °C and slowly cooled to room temperature in respective buffer conditions at the desired strand concentration for a given assay as described below. Strand concentration was calculated using the Beer–Lambert law: A = e·*C*·*l*. The extinction coefficients for each oligonucleotide were determined by the nearest neighbor method. Since some of these oligonucleotides exhibit higher-order structures, absorbance at 260 nm for all oligonucleotides was recorded at 95 °C to ensure the presence of single-stranded DNA.

### 2.2. CD Spectroscopy

Spectral and melting temperature (T_m_) analyses were conducted on a Jasco-1100 spectropolarimeter (Jasco, Easton, MD, USA) using a 1 mm optical path length quartz cell. The *CARD11* wild-type and mutant oligonucleotides were diluted to 5 µM strand concentration in 10 mM Tris-HCl buffer (pH 7.4) over a 0–100 mM range of KCl concentrations, heated at 95 °C for 5 min, and slowly cooled to room temperature overnight. Spectral data were collected over a wavelength range of 230–330 nm with a scanning speed of 100 nm/min and response time of 1 s. All spectra were recorded in triplicate, averaged, baseline corrected for signal contributions from buffers, and smoothed using the Savitzky–Golay method. Molar ellipticities for melting curves were recorded at the λ of the maximum spectra molar ellipticity. The temperature of the sample was increased at a rate of 1 °C/min from 4 °C to 95 °C. The T_m_ values were determined within ±1 °C error using the log (inhibitor) vs. response non-linear regression fit on Prism software (GraphPad v 9.0.2).

### 2.3. DNA Polymerase Stop Assay

The *CARD11* DNA polymerase stop assay sequences were annealed to a fluorescein-labeled primer (5 µM) in a 10 mM Tris-HCl buffer (pH 7.4) over a 0–100 mM range of KCl concentrations and then slow-cool annealed. This served as the substrate, which was then incubated at 200 nM in a reaction buffer of 50 mM Tris-HCl (pH 7.5), 1% glycerol, 5 mM DTT, 0.1 mg/mL BSA, and 5 mM MgCl_2_, with matching KCl concentration at room temperature for 30 min. For the compound stabilization experiments (20 mM KCl), the compounds were added to the reaction buffer and then incubated for 30 min (same 30 min). Next, Deep Vent DNA polymerase (NEB) and 250 µM dNTPs were added and primer extension was permitted at 37 °C for 30 min. The reactions were quenched with 95% formamide, 20 mM EDTA, and bromophenol blue and the mixtures were heated at 95 °C for 5 min. Samples were resolved by loading 150 fmol of DNA on a 12% polyacrylamide–7 M urea gel. The gel was visualized with a Typhoon Trio Imager (GE Healthcare, Chicago, IL, USA). The intensity of product bands was quantified using ImageQuant TL (GE Healthcare).

### 2.4. Compounds for Screening and NSC373981

The NCI Diversity Set IV of 1584 compounds was obtained from the National Cancer Institute, National Health Developmental Therapeutics Program (Bethesda, MD, USA), in the 96-well plate format. All compounds were dissolved in 100% DMSO to a 10 mM stock concentration based on the molecular weight of each compound upon receipt. Following screening and the selection of NSC373981, the compound was ordered in vial format and purity was confirmed by HPLC to be ≥95% (Supplementary Figure S1).

### 2.5. FRET Melt Screening Assay

The *CARD11* G4 FRET probe was diluted to 0.2 µM in 10 mM Tris, 20 mM KCl buffer (pH 7.4). Compounds were added to the probes at a molar equivalent of 1:5 to strand concentration (1.0 μM) and the emission of the FAM fluorophore at 520 nm was measured at each degree over the temperature range of 25–95 °C at a ramp rate of 1 °C/cycle using the BioRad CFX1000 Touch thermal cycler (Hercules, CA, USA). Buffer only and 2% DMSO treatments served as controls. The T_m_ values were determined within ±1 °C error using the log (inhibitor) vs. response non-linear regression fit on Prism software (GraphPad v 9.0.2).

### 2.6. Synthesis of Analogs

The detailed synthesis of each analog is described in Supplementary Materials. Deuterated chloroform or dimethyl sulfoxide was used as a solvent. For purity analysis, an Agilent Acquity UPLC or HPLC were used. All compounds were dissolved in methanol for purity analysis (Supplementary Figure S2). ^1^H NMR and ^13^C NMR spectra were recorded on an Agilent 400 MHz NMR spectrometer (Supplementary Figure S3).

#### 2.6.1. Synthesis of Nitro-Naphthofuran Ethanol (**R47**)

**Scheme 1:**
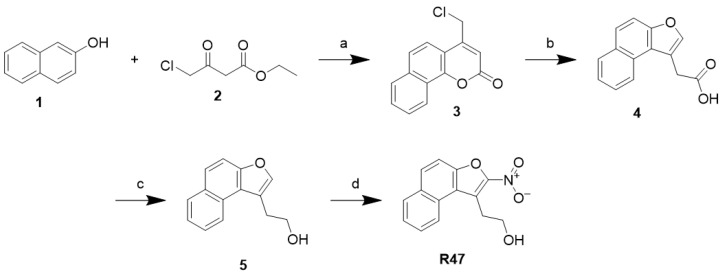
Reagents and conditions: (a) conc. H_2_SO_4_, rt; (b) 2M NaOH, 80 °C; (c) LiAlH_4_, THF, rt; (d) conc. HNO_3_, DCM, 0 °C.

The synthesis of nitro-naphthofuran ethanol **R47** has been previously described and is outlined in Scheme 1 [36]. Briefly, a Pechmann condensation was completed between naphthol (**1**) and ethyl-4-chloroacetoacetate (**2**) to yield an oxobenzopyran intermediate (**3**), which was converted to naphthofuran acetic acid (**4**) via alkaline ring contraction. The reduction of naphthofuran acetic acid by lithium aluminum hydride produced naphthofuran ethanol (**5**), and it was nitrated using nitric acid to yield nitro-naphthofuran ethanol, **R47**. 2-(2-nitronaphtho [2,1-b]furan-1-yl)ethan-1-ol (**R47**): Yield: 33%, ^1^H NMR (400 MHz, dmso) δ 8.47–8.42 (m, 1H), 8.14 (d, *J* = 9.1 Hz, 1H), 8.09 (dd, *J* = 8.1, 1.4 Hz, 1H), 7.82 (d, *J* = 9.1 Hz, 1H), 7.72 (ddd, *J* = 8.4, 7.0, 1.4 Hz, 1H), 7.61 (ddd, *J* = 8.1, 7.0, 1.1 Hz, 1H), 5.02 (s, 1H), 3.79 (t, *J* = 7.1 Hz, 2H), 3.62 (t, *J* = 7.0 Hz, 2H).^13^C NMR (101 MHz, dmso) δ 150.48, 149.64, 133.26, 131.24, 130.34, 128.81, 128.67, 126.47, 124.17, 123.41, 121.39, 112.77, 59.70, 29.54.

#### 2.6.2. Synthesis of Nitro-Naphthofuran Ethyl Amines (**R56**, **R62**, **R75**, **R76**, and **R118**)

**Scheme 2:**
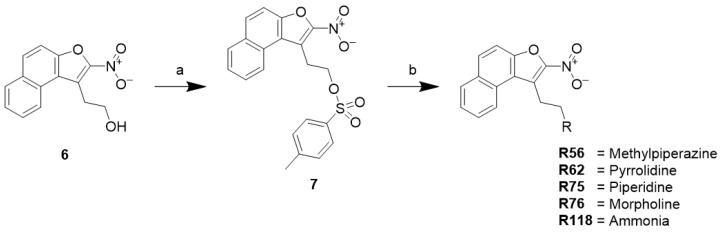
Reagents and conditions: (a) p-toluene sulfonylchloride, triethylamine, 4-dimethylaminopyridine, DCM, rt; (b) ACN, 60 °C.

The synthesis of nitro-naphthofuran amines is outlined in Scheme 2. The nitro-naphthofuran alcohol (**6**) was activated with toluene sulfonyl chloride (**7**), which was then subjected to nucleophilic replacement with different amines to generate **R56**, **R62**, **R75**, **R76**, and **R118**.

1-Methyl-4-(2-(2-nitronaphtho[2,1-b]furan-1-yl)ethyl)piperazine (**R56**): Yield: 36%, ^1^H NMR (400 MHz, CDCl_3_) δ 8.40 (d, *J* = 8.5 Hz, 1H), 8.03 (s, 1H), 8.01 (s, 1H), 7.74 (t, *J* = 7.2 Hz, 1H), 7.69–7.61 (m, 2H), 3.83–3.77 (m, 2H), 3.70–3.60 (m, 2H), 2.94–2.89 (m, 2H), 2.75 (d, *J* = 37.2 Hz, 6H), 2.45 (s, 3H). ^13^C NMR (101 MHz, cdcl3) δ 150.65, 132.63, 131.16, 130.07, 128.77, 128.40, 126.02, 124.39, 122.66, 121.20, 112.36, 56.37, 55.04, 52.92, 45.95, 23.63. *m*/*z* (ESI) calculated for C_19_H_21_N_3_O_3_ [M+H]^+^: 340.16, found: 340.44.

1-(2-(2-Nitronaphtho[2,1-b]furan-1-yl)ethyl)pyrrolidine (**R62**): Yield: 27%, ^1^H NMR (400 MHz, cdcl3) δ 8.53 (d, *J* = 8.4 Hz, 1H), 8.02 (s, 1H), 8.00 (s, 1H), 7.77 (t, *J* = 7.8 Hz, 1H), 7.64 (q, *J* = 8.2 Hz, 2H), 3.89 (t, *J* = 8.4 Hz, 2H), 3.02 (t, *J* = 8.4 Hz, 2H), 2.85 (s, 4H), 1.93 (d, *J* = 5.7 Hz, 4H). 13C NMR (101 MHz, cdcl3) δ 150.70, 132.81, 131.12, 129.97, 128.70, 126.14, 123.80, 122.90, 121.18, 112.23, 54.09, 53.99, 45.25, 29.67, 25.31, 23.53. *m*/*z* (ESI) calculated for C_19_H_21_N_3_O_3_ [M+H]^+^: 311.13, found: 311.61.

1-(2-(2-Nitronaphtho[2,1-b]furan-1-yl)ethyl)piperidine (**R75**): Yield: 65%, ^1^H NMR (400 MHz, cdcl3) δ 8.47 (d, *J* = 8.6 Hz, 1H), 8.05–8.00 (m, 2H), 8.01 (d, *J* = 1.9 Hz, 2H), 7.74 (ddd, *J* = 8.4, 7.0, 1.4 Hz, 1H), 7.67 (d, *J* = 9.1 Hz, 1H), 7.62 (ddd, *J* = 8.1, 7.0, 1.2 Hz, 2H), 3.85–3.76 (m, 2H), 2.87–2.78 (m, 2H), 2.63 (s, 4H), 1.67 (p, *J* = 5.6 Hz, 6H). 13C NMR (101 MHz, cdcl3) δ 150.67, 132.62, 131.15, 130.01, 128.84, 128.39, 125.99, 124.82, 122.81, 121.35, 112.36, 57.20, 54.40, 26.00, 24.28, 23.57. *m*/*z* (ESI) calculated for C_19_H_20_N_2_O_3_ [M+H]^+^: 325.15, found: 325.38.

4-(2-(2-Nitronaphtho[2,1-b]furan-1-yl)ethyl)morpholine (**R76**): Yield: 53%,^1^H NMR (400 MHz, cdcl3) δ 8.43 (d, *J* = 8.3 Hz, 1H), 8.05–8.01 (m, 2H), 7.74 (ddd, *J* = 8.4, 7.0, 1.4 Hz, 1H), 7.68 (d, *J* = 9.1 Hz, 1H), 7.63 (ddd, *J* = 8.1, 7.0, 1.1 Hz, 1H), 3.84–3.77 (m, 6H), 2.89–2.84 (m, 2H), 2.67 (t, *J* = 4.8 Hz, 4H). 13C NMR (101 MHz, cdcl3) δ 150.70, 132.70, 131.20, 130.12, 128.78, 128.43, 126.06, 124.24, 122.62, 121.20, 112.41, 66.95, 56.86, 53.58, 29.69, 23.51. *m*/*z* (ESI) calculated for C_18_H_18_N_2_O_4_ [M+H]^+^: 326.13, found: 326.29.

2-(2-Nitronaphtho[2,1-b]furan-1-yl)ethan-1-amine (**R118**): Yield: 31%, ^1^H NMR (400 MHz, cdcl3) δ 8.38 (d, *J* = 8.4 Hz, 1H), 8.05–7.88 (m, 2H), 7.76–7.55 (m, 3H), 3.76 (t, *J* = 7.2 Hz, 2H), 3.31 (t, *J* = 7.3 Hz, 2H). 13C NMR (101 MHz, cdcl3) δ 150.80, 132.78, 131.23, 130.11, 128.86, 128.46, 126.07, 123.97, 122.72, 121.25, 112.37, 29.68, 29.64, 29.60. *m*/*z* (ESI) calculated for C_14_H_12_N_2_O_3_ [M+H]^+^: 257.08, found: 257.51.

#### 2.6.3. Synthesis of Halogenated Naphthofuran Ethanol (**R67** and **R68**)

**Scheme 3:**
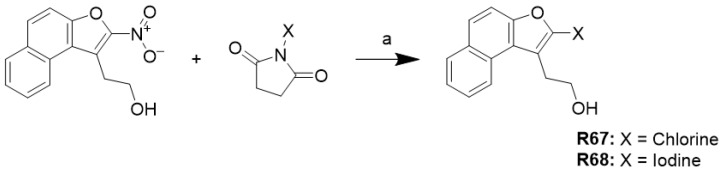
Condition: (a) Hexafluoroisopropanol, 55 °C.

The synthesis of halogenated naphthofuran ethanol is shown in Scheme 3. The nitro compound **R47** was treated with chlorine or iodine succinimide using hexafluoroisopropanol as solvent to yield **R67** and **R68**.

2-(2-Chloronaphtho[2,1-b]furan-1-yl)ethan-1-ol (**R67**): Yield: 57%, ^1^H NMR (400 MHz, cdcl3) δ 8.28–8.23 (m, 1H), 7.96–7.90 (m, 1H), 7.70 (d, *J* = 8.7 Hz, 1H), 7.62–7.55 (m, 2H), 7.49 (ddd, *J* = 8.1, 6.9, 1.2 Hz, 1H), 4.02 (t, *J* = 7.2 Hz, 2H), 3.30 (t, *J* = 6.9 Hz, 2H). 13C NMR (101 MHz, cdcl3) δ 151.26, 130.78, 129.26, 127.56, 126.72, 125.63, 124.65, 122.70, 121.85, 113.07, 111.85, 61.54, 28.63.

2-(2-Iodonaphtho[2,1-b]furan-1-yl)ethan-1-ol (**R68**): Yield: 78%, ^1^H NMR (400 MHz, dmso) δ 8.31 (d, *J* = 8.4 Hz, 1H), 8.02 (d, *J* = 8.3 Hz, 1H), 7.77 (d, *J* = 9.0 Hz, 1H), 7.74 (d, *J* = 9.0 Hz, 1H), 7.64 (ddd, *J* = 8.4, 6.8, 1.3 Hz, 1H), 7.52 (t, *J* = 7.5 Hz, 1H), 4.97 (s, 1H), 3.65 (t, *J* = 7.5 Hz, 2H), 3.09 (t, *J* = 7.5 Hz, 2H). 13C NMR (101 MHz, dmso) δ 156.20, 130.68, 129.48, 127.35, 126.93, 125.94, 125.25, 125.11, 123.22, 121.56, 112.45, 102.15, 60.46, 31.44.

#### 2.6.4. Synthesis of Methyl Pyrazole Nitro Naphthol Ethanol (**R114**)

**Scheme 4:**
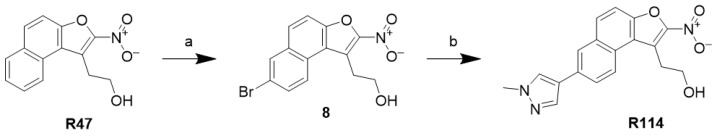
Reagents and conditions: (a) Br_2_, acetic acid; (b) 1-methyl-4-pyrazole boronic acid pinacol ester, K_2_CO_3_, Pd_2_(PPh_3_)_43_, 4:1 DMF: H2O, 90 °C.

The synthetic scheme is outlined in Scheme 4. **R47** was brominated using bromine to generate the halogenated intermediate (**8**). Methyl pyrazole was then coupled to **8** using Suzuki coupling to generate **R114**.

2-(7-(1-Methyl-1H-pyrazol-4-yl)-2-nitronaphtho[2,1-b]furan-1-yl)ethan-1-ol (**R114**): Yield: 56%, ^1^H NMR (400 MHz, cdcl3) δ 8.37 (d, *J* = 8.4 Hz, 1H), 8.01–7.95 (m, 2H), 7.98–7.91 (m, 1H), 7.68 (d, *J* = 8.9 Hz, 1H), 7.64–7.55 (m, 1H), 7.51–7.43 (m, 1H), 4.49 (t, *J* = 7.7 Hz, 2H), 4.00 (s, 3H), 3.50 (t, *J* = 7.7 Hz, 2H). *m*/*z* (ESI) calculated for C_18_H_15_N_3_O_4_ [M+H]^+^: 338.11, found: 338.59.

#### 2.6.5. Synthesis of Nitro-Naphthofuran (**R158**)

**Scheme 5:**
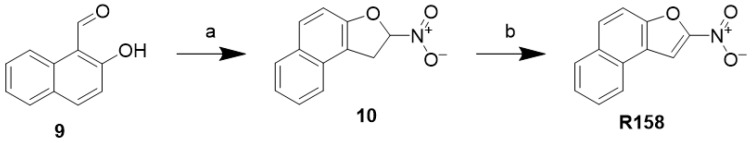
Reagents and conditions: (a) bromonitromethane, K_2_CO_3_, acetone, rt; (b) acetic anhydride, reflux.

Synthesis of nitro-naphthofuran compound is outlined in Scheme 5. Bromontiromethane was added to reacted with naphthaldehyde (**9**) to generate intermediate (**10**). Following this, **10** was refluxed in acetic anhydride to generate nitro-naphthofuran **R158**.

2-Nitronaphtho[2,1-b]furan (**R158**): Yield: 29%, ^1^H NMR (400 MHz, CDCl_3_) δ 8.18 (dd, *J* = 8.1, 4.5 Hz, 2H), 8.02 (t, *J* = 8.5 Hz, 2H), 7.75–7.69 (m, 2H), 7.63 (ddd, *J* = 8.2, 7.1, 1.2 Hz, 1H). ^13^C NMR (101 MHz, dmso) δ 152.66, 151.87, 132.60, 130.79, 129.68, 128.66, 128.08, 126.95, 124.41, 122.59, 112.86, 108.91.

### 2.7. Cell Lines

The ABC DLBCL cell lines used in the study were RIVA and HBL1. RIVA (ACC585) was purchased from the DSMZ repository and HBL1 was a gift from the laboratory of Dr. Lisa Rimsza. The GCB DLBCL cell lines, HT (CRL2260) and VAL (ACC586), were purchased from the American Type Culture Collection and DSMZ, respectively. The benign GC B cells, GM22671, were obtained from the Coriell Institute for Medical Research (Camden, NJ, USA). All cells were cultured in 10% fetal bovine serum (Atlanta Biologicals, Flowery Branch, GA, USA) and 1% penicillin/streptomycin (Gibco, Grand Island, NY, USA) supplemented RPMI 1640 medium (Corning, Corning, NY, USA). Cell viability was assessed by trypan blue exclusion, and the experiments were conducted when viability ≥ 90%. All cell lines were tested for mycoplasma every 6 months and were negative prior to and for the duration of this study. All cell lines were authenticated by the University of Arizona Genetics Core (Tucson, AZ, USA) using the PowerPlex 16 System (Promega, Madison, WI, USA), which consists of forensic-style 15 autosomal STR loci, including 13 CODIS DNA markers (nine of the standard loci collected by ATCC and DSMZ), and amelogenin every 12 months (Supplementary Table S2).

### 2.8. Cytotoxicity Assay

The concentration at which growth was 50% of untreated and 0.4% DMSO vehicle treated controls for each compound (GI_50_) was determined by the 3-(4,5-dimethylthiazol-2-yl)-5-(3-carboxymethoxyphenyl)- 2-(4-sulfophenyl)-2H-tetrazolium (MTS) colorimetric assay, as per the manufacturer’s specifications (Promega, Madison, WI, USA). Cells were plated in a 96-well plate at a concentration of 20,000 cells/well and were equilibrated overnight at 37 °C. Cells were then treated over a dose–response range of 0.02–40 µM, at 2-fold dilutions. The cells were incubated with each compound for 72 h, subsequently incubated for an additional 3 h with the MTS reagent, and assayed for absorbance at 490 nm on the BioTek Epoch plate reader (Santa Clara, CA, USA). The cell viabilities were logarithmically transformed and non-linear curve fitted using least squares regression, and the GI_50_ values were calculated using GraphPad Prism software version 9.0.2 (GraphPad Software).

### 2.9. Gene Expression by Real-Time Quantitative PCR

The effects of NSC373981 on *CARD11* gene expression were determined using real-time quantitative PCR as previously described. Cells seeded at a density of 250,000 cells/mL were harvested following 24 h treatment with NSC373981 (2.5, 5.0, and 10.0 µM). Cells untreated and treated with 0.1% DMSO were used as controls. Total RNA was isolated with a Roche High Pure RNA isolation kit (San Francisco, CA, USA) and reverse transcription was performed using the BioRad iScript kit according to the manufacturer’s protocols. Real time quantitative PCR was conducted with the BioRad Probe Supermix and TaqMan Probes using the BioRad CFX1000 Touch thermal cycler. The Ct values were normalized to TATA-binding protein (*TBP*; Hs00427620_m1) and compared to the untreated controls to obtain ΔΔCt values. The TaqMan probes for genes of interest were as follows: *CARD11*, Hs01060620_m1; *BCL2*, Hs 00608023_m1; *MYC*, Hs00153408_m1; *KRAS*, Hs00364284_g1; and *TERT*, Hs00972650_m1.

### 2.10. Statistical Analysis

Data are presented as representative from three independent experiments and/or as the mean ± standard error from at least three independent experiments unless otherwise stated. Significance (*p* < 0.05) was evaluated using either a Two-Way ANOVA with the Tukey’s multiple comparisons test or a One-Way ANOVA with Dunnett’s multiple comparisons test. All analyses were performed with GraphPad Prism software version 9.0.2.

## 3. Results

### 3.1. G-Rich Element within CARD11 Promoter Forms a Stable G4 Structure

**Figure 1:**
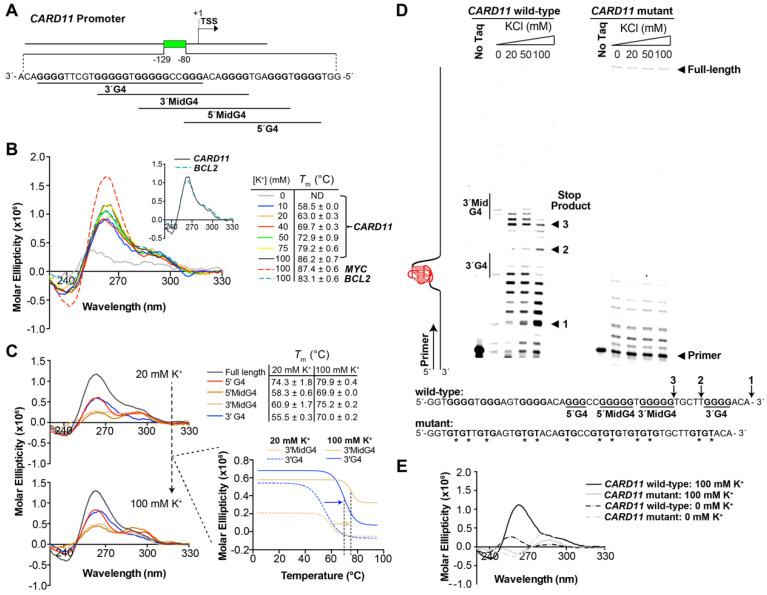
G-rich sequence in the *CARD11* promoter regions forms G-quadruplex (G4) structures. (**A**) Schematic of the *CARD11* promoter region with the potential G4 forming elements (green box) identified in close proximity to the transcription start site (TSS). (**B**) Circular dichroism (CD) spectra of the *CARD11* G4 sequence in the absence and presence of increasing concentrations of KCl. Corresponding melting temperatures (T_m_) are provided. CD spectra of the established *MYC* (dashed red line) and *BCL2* (dashed blue line) G4 forming sequences in 100 mM KCl served as controls. Inset, direct comparison of the *CARD11* and *BCL2* G4 sequence spectra at 100 mM KCl. Data represent three independent experiments. (**C**) CD spectra of the *CARD11* G4 full-length, 5′ end, 5′ mid, 3′ mid, and 3′ end runs of Gs in the presence of 20 and 100 mM KCl. Corresponding T_m_ are provided with the shifts in melt curves for the 3′ mid and 3′ end G4s, which displayed the largest increases. Data represent two independent experiments. (**D**) Representative DNA polymerase stop assay gel of three independent experiments (see Supplementary Figure S4 for replicates and Supplementary Figure S5 for base-resolution in unadjusted gels) of the *CARD11* G4 wild-type and mutant sequences in the absence and presence of increasing concentrations of KCl. * denotes the thymine substituted guanines. (**E**) CD spectra of the *CARD11* G4 wild-type and mutant sequence with thymine substitutions for Gs in each run in the absence or presence of 100 mM KCl.

We discovered a G-rich sequence 80 bases upstream of the *CARD11* transcription start site (TSS) consisting of 49 bases on the noncoding strand (Figure 1A) that is capable of folding into a stable G4 structure (Figure 1B). This purine dense element has a stretch of seven runs of 3 to 5 Gs with potential to form multiple G4 structures (Figure 1A,C,D), as often seen in G4 sequences that contain more than the minimum four runs of Gs [37,38]. The G4 signature of the full-length *CARD11* oligomer demonstrates the typical K^+^-dependent CD spectra with a corresponding increase in thermal stability reflected in the melting temperature (T_m_) that is comparable to the well-characterized *MYC* and *BCL2* G4s (Figure 1B). The presence of a shoulder in the spectra around 290 nm indicates the *CARD11* G4 most likely adopts a mixed antiparallel–parallel conformation and is supported by the spectral overlap with the *BCL2* G4, which is known to exhibit the same folding pattern [37] (Figure 1B, inset). We then evaluated the ability of truncated *CARD11* G4 sequences based on four consecutive G runs from the 5′ end, 5′ middle (mid), 3′ mid, and 3′ end of the full-length to form G4 structures. We observed that 5′ end and 3′ mid G4s displayed the highest thermal stability at both low and high K^+^ concentrations; however, the 3′ end G4 was substantially stabilized at 100 mM K^+^ with a 15.5 °C shift in T_m_ from 20 mM K^+^ (Figure 1C). We confirmed that the *CARD11* oligomer forms stable G4s using the DNA polymerase stop assay where the ability of a polymerase to traverse a linear piece of DNA to produce a full-length product is leveraged to observe shorter, terminated products when the polymerase encounters a G4 and cannot continue to synthesize the DNA from the template. We observed three major stop products for the *CARD11* wild-type sequence with increasing KCl concentrations, which most likely represent 3′ end, 3′ mid, and 5′ mid G4s (Figure 1D). The prominent, KCl-dependent 3′ end stop product is consistent with the marked increase in stability of the 3′ end G4 observed in the T_m_ analysis upon increasing K^+^ from 20 mM to 100 mM (Figure 1C). Similarly, 3′ mid G4 exhibited a notable shift in T_m_ (Δ14.3 °C) and a corresponding increase in a stop product near the presumed location of this G4 (stop product 2; Figure 1D, Supplementary Figure S4A). With the increase in stop products 1 and 2 at higher K^+^, there was a reduction in stop product 3 that was prominent at lower K^+^ (Figure 1D). As expected, based on the inability of the *CARD11* mutant, a sequence where at least one G from each of the seven runs was substituted with a thymine to form a G4 structure according to CD spectroscopy (Figure 1E), and there were no distinct stop products formed and a full-length product is visible even at high KCl concentrations.

### 3.2. A CARD11 G-Quadruplex Stabilizing Small Molecule Inhibits Transcription

**Figure 2:**
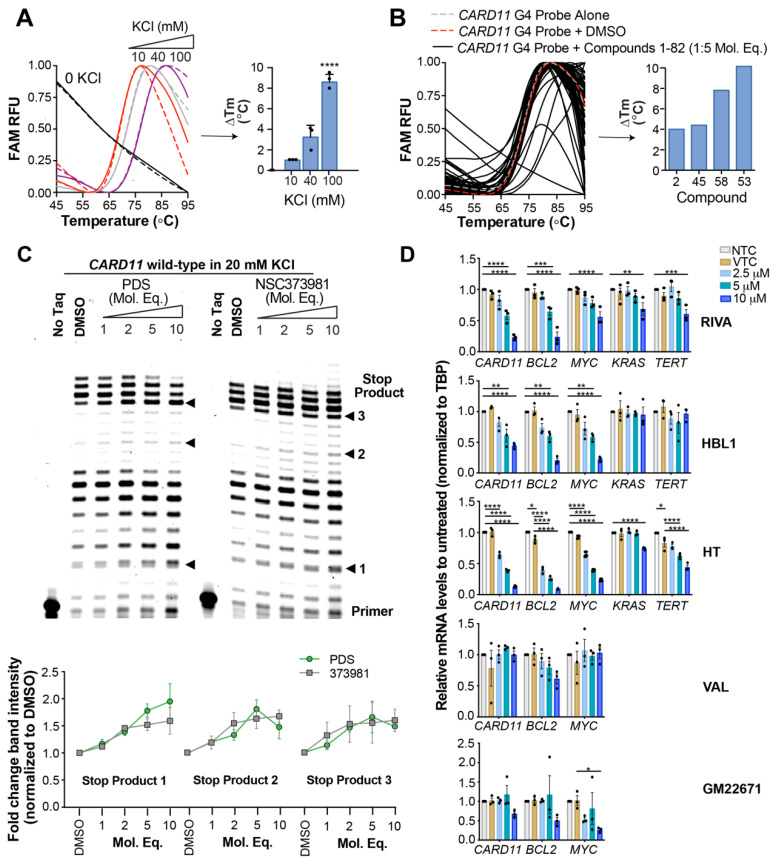
Identification of a *CARD11* G-quadruplex (G4) stabilizing small molecule that downregulates *CARD11* gene expression. (**A**) Representative FRET melt curves of the *CARD11* G4 fluorescent probe in 10, 40, or 100 mM KCl relative to a KCl free buffer and the corresponding differences in T_m_ (ΔT_m_). Data represented as mean ± SE from three independent experiments. (**B**) Representative FRET melt curves of a compound plate and the corresponding ΔT_m_ relative to probe with DMSO in the presence of 5 molar equivalent concentrations of candidate compounds. Compounds producing a right shifted melt curve from the probe alone with DMSO vehicle control indicating an increase in T_m_ of at least 4 °C were considered candidates. Screen was performed with probes in a 40 mM KCl buffer. (**C**) Representative DNA polymerase stop assay gel of three independent experiments (see Supplementary Figure S11 for replicates) of the *CARD11* wild-type sequence in 20 mM KCl in the presence of increasing molar equivalents of pyridostatin (PDS) or NSC373981. DMSO served as the vehicle control. Quantification is shown below as fold-change in band intensity normalized to DMSO. (**D**) Real-time quantitative PCR for *CARD11*, *BCL2*, MYC, *KRAS*, and *TERT* mRNA levels in diffuse large B cell lymphoma (DLBCL) and benign B cells following treatment with increasing concentrations of NSC373981 for 24 h. Untreated and DMSO treated cells served as controls. Genes of interest were normalized to the TBP housekeeping gene. Data represented as mean ± SE from three independent experiments. * *p* < 0.05; ** *p* < 0.01; *** *p* < 0.001; **** *p* < 0.0001.

We performed a fluorescent-based compound screen of the NCI Diversity Set IV library of 1584 small molecules to identify *CARD11* G4 interactive molecules. In using the established FRET-melt assay, we can detect shifts in G4 T_m_ under different G4-forming conditions. In this experiment, T_m_ is calculated as the temperature at which half of the fluorescence emitted from the donor fluorophore (FAM) is lost to the acceptor fluorophore (TAMRA) upon G4 formation where these fluorescent molecules are brought within close proximity of one another and energy is transferred [39]. The *CARD11* G-quadruplex forming sequence labeled with FAM and TAMRA fluorophores at the 5′- and 3′- ends, respectively, served as molecular bait for identifying small molecules that stabilized the structures, resulting in a detectable increase in T_m_. Before conducting the compound screen, we verified the FRET probe consisting of the *CARD11* G4 sequence folded into a KCl-dependent G4 structure (Figure 2A). We then screened the compounds in a 96-well plate format with the same controls, *CARD11* G4 probe in 40 mM KCl buffer with and without 2% DMSO (Figure 2B). Hits were defined as shifting the T_m_ by at least 4 °C and we obtained 21 compounds producing a 1.3% hit rate. A candidate compound, NSC373981, was selected and confirmed to stabilize the *CARD11* G4 by DNA polymerase stop assay in a dose-dependent manner similar to a known pan-G4 ligand, pyridostatin (PDS) (Figure 2C). Of note, starting at 1:2 molar equivalents of DNA:compound for both PDS and NSC373981, there was an increase in stop product 1 relative to DMSO vehicle treated control, as seen in the previous KCl stabilization of the *CARD11* G4 (Figure 1D, Supplementary Figure S4A), although incubation with PDS resulted in a quantifiable higher band intensity of stop product 1 relative NSC373981 at 1:5 and 1:10 molar equivalents (Figure 2C, bottom panel).

Historically, G4s are regarded as transcriptional silencers, but there is recognition that strand location impacts the regulatory role of these structures in inhibiting or promoting transcription [28,30,40,41,42,43,44,45,46]. Since the *CARD11* G4 sequence resides on the non-coding, template strand, we hypothesized that G4 stabilization could result in transcription silencing. Consistent with previous work for some non-coding strand G4s [22,30,37,46], we observed a significant, dose-dependent repression of *CARD11* mRNA levels in both ABC DLBCL cells, RIVA and HBL1, and the GCB DLBCL cell HT following NSC373981 treatment (Figure 2D). In contrast, the treatment of the GCB DLBCL cell VAL and the benign GC B cells, GM22671, with NSC373981 did not result in any significant change in *CARD11* mRNA levels. Considering the prevalence of different G4 forming sequences in other important cancer initiating and driving genes [26,44], we determined whether NSC373981 preferentially targets the *CARD11* G4 for transcription regulation relative to other promoter G4s. When we observed *CARD11* downregulation, we detected a concomitant dose-dependent decrease in mRNA for *BCL2* and *MYC* genes (Figure 2D). Given that the *CARD11* G-rich sequence displays a similar G4 signature to *BCL2* (Figure 1B) and NSC373981 increased the *BCL2* G4 melting temperature (Supplementary Figure S6) as well as a predicted binding of NSC373981 with the *MYC* G4 through Pi–Pi stacking interactions [47,48] (Supplementary Figure S7); most likely, this simultaneous lowering of *BCL2* and *MYC* occurs through promiscuous G4 stabilization. However, it is important to note that, due to the *CARD11* activation of NFkB, a loss of *CARD11* may also indirectly restrict *BCL2* and *MYC* expression, which are known downstream targets of NFkB and BCR signaling [49,50,51,52]. Additionally, we examined mRNA levels of *KRAS* and *TERT* as representatives of well-characterized promoter G4s with tandem structures and atypical folding patterns [29,32,53,54]. In the same treatments with NSC373981, two of the three cell lines with decreased *CARD11* mRNA (RIVA and HT) display a 25–30% decrease in *KRAS* or a 40–55% in *TERT* whereas the other DLBCL cell (HBL1) did not have detectable differences in either *KRAS* or *TERT* mRNA (Figure 2D). This effect on *KRAS* and *TERT* mRNA expression often required the highest NSC373981 concentration and appears less extensive compared to the 45–90% lowering of *CARD11*, *BCL2*, and *MYC* mRNA, suggesting a slight preference for these more canonical G4 regulated genes. Of note, RIVA and HT, with the expression of all five oncogenes affected by NSC373981 treatment, exhibited a higher cytotoxicity relative to HBL1 cells (Supplementary Figure S8A,D). Despite little to no change in *CARD11* mRNA in the benign B cells (GM22671), NSC373981 was cytotoxic at low µM concentration, which could be attributed to the potential of the nitro group to generate oxygen radicals within a cellular context due to the electron-withdrawing effect that can cause DNA damage. Cytotoxicity did not correlate with the basal expression of *CARD11*, since VAL and HBL1 cells exhibit similar GI_50_ concentration, but a 2-fold difference in basal levels (Supplementary Figures S8 and S9). Likewise, RIVA, HT, and GM22671 cells express the same basal level of *CARD11* as VAL, and yet they are more sensitive to NSC37981 (Supplementary Figures S8 and S9).

In support of G4 stabilization as the main driver for the mRNA repression of NSC373981, parallel concentrations of PDS induced a similar significant, dose-dependent reduction in *CARD11* mRNA with decreased levels of *BCL2*, *MYC*, and *TERT* mRNA (Supplementary Figure S10). However, unlike the low micromolar cytotoxicity of NSC373981 in cells with repressed expression of all five oncogenes (RIVA and HT), PDS exhibited a higher GI_50_ by at least 5-fold (Supplementary Figure S8B,D).

### 3.3. Naptho(2,1-b)furan-1-ethanol,2-nitro- Requires the Nitro Group for G-Quadruplex Stabilization and CARD11 Gene Repression

**Figure 3:**
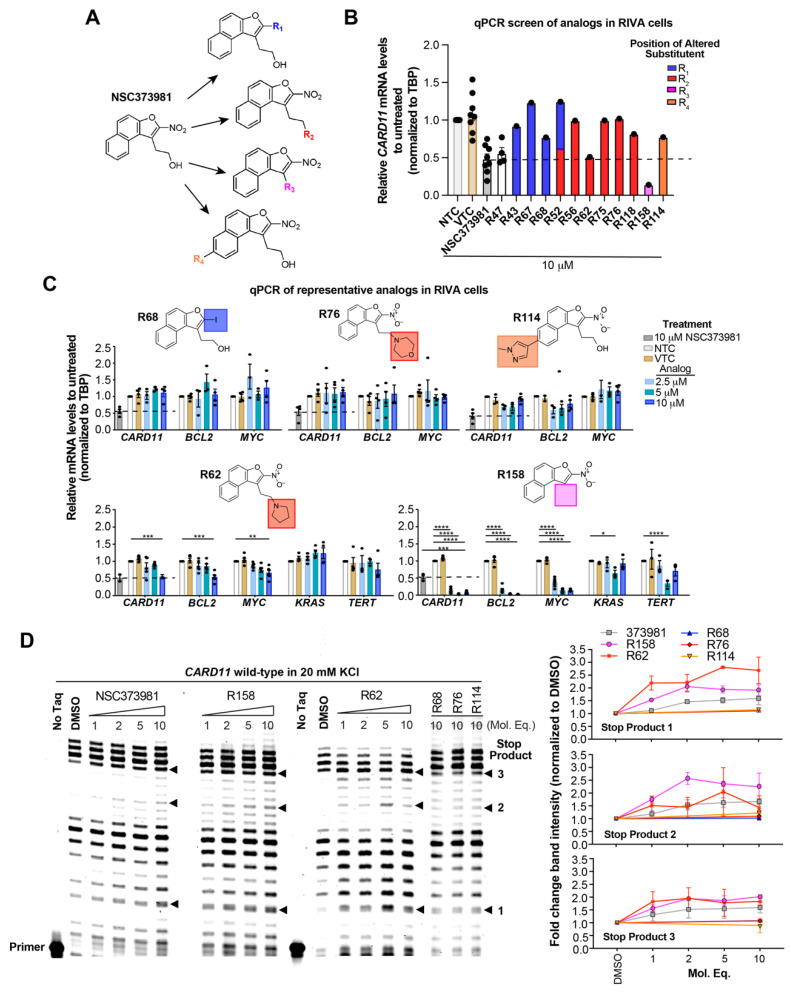
NSC373981 requires nitro-group for *CARD11* G-quadruplex (G4) stabilization and repression of *CARD11* transcription. (**A**) Schematic depicting the NSC373981 parent compound structure and analogs generated to alter 4 different positions. (**B**) qPCR screen of 11 analogs in RIVA ABC diffuse large B cell lymphoma (DLBCL) cells following 24 h treatment. Untreated (NTC, no treatment control) and DMSO (VTC, vehicle treatment control)-treated cells served as controls. Data from controls represented as mean ± SE from 4 to 8 independent experiments. Data from 11 analogs represent a single experiment. (**C**) qPCR of RIVA cells treated for 24 h at increasing concentrations of the respective analog shown in the schematic with the specific altered substituent. NTC and VTC were used as negative controls and NSC373981 at 10 µM served as a positive control. Data represented as mean ± SE from three independent experiments. (**D**) Representative DNA polymerase stop assay gel of two independent experiments (see Supplementary Figure S12 for replicate gel) With the quantification of band intensities of the *CARD11* wild-type sequence in the presence of increasing molar equivalents of NSC373981, **R62**, and **R158** and 1:10 molar equivalent of **R68**, **R76**, and **R114** compared to DMSO control. * *p* < 0.05; ** *p* < 0.01; *** *p* < 0.001; **** *p* < 0.0001.

With the long-term goal of targeting the *CARD11* G4 as an alternative therapy for DLBCL and to identify what substituents of NSC373981 are important for the G4-interactive, *CARD11* mRNA-repressive pharmacophore for developing lead molecules, we generated 11 analogs based on altered groups in four different positions, R_1_–R_4_ (Figure 3A, Table 1). The syntheses of these analogs are outlined in Scheme 1, Scheme 2, Scheme 3, Scheme 4 and Scheme 5 and provided in more detail in the Supplementary Materials. Initially, we performed a qPCR screen of the 11 analogs and the synthesized version of NSC373981, **R47** using RIVA cells following a 24 h treatment at 10 µM, the highest concentration in which we see the greatest effect of NSC373981. We confirmed **R47** achieved comparable downregulating effects of *CARD11* mRNA as NSC373981, which provided confidence in accurate synthesis of analogs using the napthofuran 3-membered ring backbone (Figure 3B). The first generated analog was **R43**, where removal of the nitro group located at the R_1_ position abolished *CARD11* mRNA repression activity (Figure 3B). We next tested whether substitution at the R_1_ position would recover the transcriptional effect by using Cl (**R67**) or I (**R68**); however, neither halogen restored the repressive activity to the same level as NSC373981 (Figure 3B). We confirmed the loss of *CARD11* activity for **R68** over a concentration gradient and demonstrated that there was also no effect on *BCL2* and *MYC* mRNA levels (Figure 3C). Additionally, **R68** did not stabilize *CARD11* G4 as indicated by the absence of increased stop products in the DNA polymerase assay (Figure 3D). Taken together, the null effects of **R43**, **R67**, and **R68** on mRNA expression and lack of G4 stabilization of **R68** suggests that the nitro group plays a role in NSC373981 acting as a *CARD11* G4 stabilizer and downregulating *CARD11* expression.

While leaving the nitro group in place, we then generated analogs with amine substitutions of the hydroxyl group (-OH) at R_2_. Of these five analogs, **R62** with a pyrrolidine, the only five-membered ring amine substituent, exhibited a similar reduction in *CARD11* mRNA as NSC373981 (Figure 3B), which was validated and shown to extend to *BCL2* and *MYC* repression but not *KRAS* or *TERT* (Figure 3C). **R62** retained *CARD11* G4 stabilization activity with prominent polymerase arrest at stop product 1, particularly for 1:5 molar equivalents (Figure 3D), suggesting that the -OH substituent has potential to improve selectivity. In contrast to **R62**, substituting -OH with a six-membered ring amine (**R56**, **R75**, **R76**) or with a basic amino group -NH_2_ (**R118**) attenuated transcription repression activity (Figure 3B). As a representative, **R76** was confirmed to have no effect on mRNA expression of *CARD11*, *BCL2*, or *MYC* (Figure 3C) or on *CARD11* G4 stabilization (Figure 3D). In addition, we removed the nitro group from **R56** (**R52**), which also generated a -OH substituted counterpart to **R43**, and as expected, **R52** did not induce a lowering of *CARD11* mRNA (Figure 3B). Next, we examined the effects of preserving the nitro group while removing the entire ethanol side chain (**R158**). **R158** induced a significant and almost complete knock-down of *CARD11* mRNA relative to NSC373981 (Figure 3B,C). This potent transcriptional inhibition was also observed for *MYC* and *BCL2*, and although it is not as dramatic, there was a decrease in *KRAS* and *TERT* levels indicating that activity is enhanced in the absence of a substituent at R_3_. **R158** stabilized not only the *CARD11* G4 with notable increases in stop products 1 and 2 (Figure 3D) but elevated the melting temperature of the *BCL2* G4 by 10 °C, whereas NSC373981 produced a shift of 4 °C (Supplementary Figure S6). Lastly, to evaluate how aromatic substitution on the other end of molecule affects *CARD11* G4 stabilization and subsequent transcription, we generated **R114**. The addition of a methyl pyrazole to the R_4_ position failed to produce an active compound. **R114** did not alter *CARD11*, *BCL2*, or *MYC* mRNA levels (Figure 3B,C) and did not stabilize *CARD11* G4, as indicated by the absence of enhanced stop products in the DNA polymerase assay (Figure 3D). Overall, the removal of the nitro group or substitutions of -OH at the R_2_ position, with the exception of pyrrolidine (**R62**), compromised the *CARD11* G4 stabilization and mRNA downregulation activity of NSC373981, suggesting that both the presence of the nitro group and a fairly compact substituent at R_2_ are critical for preserving activity. Despite the augmented potency of **R158**, the preferential stabilization of *BCL2* G4, along with the lack of anti-*KRAS* and *TERT* activity of **R62** points toward the importance of a substituent at the positions of the ethanol side chain and the -OH to limit interaction with other G4 structures. Furthermore, the cytotoxicity of the representative analogs appeared to correlate with activity in that inactive analogs **R68** and **R114** displayed at minimum a 6-fold less toxicity, while analogs with enhanced G4 stabilization, **R158**, or comparable activity, **R62**, induced corresponding 5-fold higher or similar cytotoxicity as NSC373981, respectively (Supplementary Figure S8C,D). The increase in GI_50_ for **R62** in RIVA and HT to a similar concentration as seen in HBL1, which remained unchanged from NSC373981, is consistent with the abrogated effect on *KRAS* and *TERT* mRNA. Interestingly, **R76**, an inactive analog, was cytotoxic within the same range as NSC373981 and **R62** (Supplementary Figure S8C,D).

## 4. Discussion

This study is the first to not only demonstrate that the *CARD11* promoter has a G-rich sequence capable of folding into a G4 structure but that this structure is targetable for the stabilization and downregulation of *CARD11* transcription. Additionally, we found that there are potentially at least four different G4s that can form from the full-length sequence with the 5′ end and 3′ mid four G runs providing the most thermally stable structures. While the current evidence regarding strand bias for whether a G4 structure acts as a silencer or activator of transcription remains mixed and strongly supports the possibility that the function of the G4 may be dependent on promoter contexts including parameters such as distance from TSS and presence or absence of transcription repressor and activator proteins [28,30,40,41,42,43,44,45,46,55], our data validate that G4s located on the non-coding strand tend to act as transcription silencers. We show that both a known pan-G4 ligand, PDS, and the newly identified small molecule NSC373981 from the NCI diversity library stabilized *CARD11* G4 and consequently lowered *CARD11* mRNA expression. Interestingly, of the five cells tested, the VAL DLBCL cell line and GM22671 benign GC B cells did not exhibit *CARD11* repression following treatment with NSC373981. This lack of response is unlikely due to a difference in basal *CARD11* expression since responsive lines, RIVA and HT, have similar baseline *CARD11* mRNA levels. It is possible the VAL and GM22671 cells do not contain an intact *CARD11* G-rich sequence; however, sequencing the promoter region was beyond the scope of this initial study.

When there was a reduction in *CARD11*, we observed a concomitant decrease in levels of both *BCL2* and *MYC* mRNA. We found that the *CARD11* G4 sequence shares a similar signature CD spectrum with *BCL2* for a mixed antiparallel–parallel structure and NSC373981 also increases *BCL2* G4’s melting temperature. Taken together with a predicted interaction with the *MYC* G4, NSC373981 G4 targeting activity extends beyond the *CARD11* promoter. The desired outcome of inhibiting *CARD11* is to block BCR signaling, which culminates in the activation of NFkB [33,34,35] and turning on the expression of downstream prosurvival effector molecules including *BCL2* and *MYC* [49,50,51]. Thus, by lowering *CARD11*, we may also indirectly inhibit additional transcription of *BCL2* and *MYC*. In addition to repressing *CARD11*, *BCL2*, and *MYC*, the expressions of two other G4-driven genes, *KRAS* and *TERT*, were often lowered by NSC373981. Since achieving selectivity for a particular G4 structure is an important milestone in the field of molecularly targeting G4 structures, we generated analogs to provide understanding into the pharmacophore required for *CARD11* G4 interaction and stabilization while improving pharmacodynamic properties.

The analogs were synthesized based on the accessible sites on the furan ring core of NSC373981. When the nitro group in the parent compound was removed or replaced with a less mutagenic halogen (R_1_ position: **R43**, **R67**, **R68**), *CARD11* G4 stabilization and the effect on *CARD11* mRNA were completely lost, indicating the nitro group is necessary for inducing repression of *CARD11*. The iodine and chlorine substitutions in **R67** and **R68** are both weakly electron withdrawing when compared to the nitro group found in **R47** (synthesized NSC373981). In the second set of analogs, the -OH group was replaced with various amines (R_2_ position analogs) to improve the solubility of the furan ring core. Replacing the -OH group with a primary amine (**R118**) only reduced *CARD11* mRNA expression by ~20% in the screen, suggesting more than one acidic proton (-OH has one acidic proton and NH_2_ has two) or an aliphatic, and basic amine is not preferred for transcription inhibition even though the amine is readily ionizable to help improve solubility. All four analogs with a heterocyclic amine with six-membered ring R_2_ substitution (**R52**, **R56**, **R75**, and **R76**) did not alter *CARD11* mRNA levels. Of note, **R62**, a substituted amine with a five-membered ring, induced a repression of *CARD11* comparable to that of **R47** and NSC373981 with a diminished effect on *KRAS* and *TERT* and maintained *CARD11* G4 stabilization activity, indicating that this substituent functions not only in activity but also possibly in selectivity. Removing the ethanol side chain altogether from the furan ring (**R158**) resulted in a dramatic increase in potency for inhibiting *CARD11* transcript production and enhanced G4 stabilization. However, **R158** significantly reduced *BCL2*, *MYC*, *KRAS*, and *TERT* with a notable stabilization of the *BCL2* G4, further supporting the alcohol side chain and -OH group provides preferential targeting toward *CARD11* G4. These pharmacological insights, while limited due to the relatively small number of analogs, set the foundation for continued drug development and design of *CARD11* G4 interactive molecules.

In demonstrating the presence of a G4 forming sequence in a key BCR signaling factor, our findings strongly suggest that this DNA motif plays a critical role in regulating the expression of the *CARD11* oncogene and has the potential to serve as a molecular target. An aggressive subset of DLBCL relies on BCR signaling, which is a pathway highly sought after for targeted therapy. Thus far, there is minimal success, with the major obstacle facing in most approaches being the cancer precision medicine of inherent or acquired resistance due to alterations in the protein–drug interface [14,56,57]. We propose that targeting the G4 DNA secondary structure within *CARD11* may overcome this mechanism of drug resistance by directly limiting the amount of gene available for translation into protein. Our study provides the groundwork for further exploring the predominant, biologically relevant folding pattern of the *CARD11* G4 structure and how targeting this structure impacts BCR signaling for developing new therapeutic strategies in BCR-dependent lymphomas.

## Figures and Tables

**Table 1:** Chemical structure and molecular weight of NSC373981 analogs.

Name	Analog Structure	Analog R-Position	Molecular Weight (g/mol)
**R47**	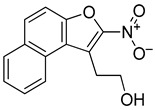	Synthesized parent compound, NSC373981	257.24
**R43**	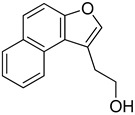	R_1_	212.24
**R67**	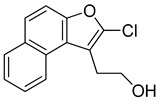	R_1_	246.69
**R68**	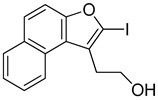	R_1_	338.14
**R52**	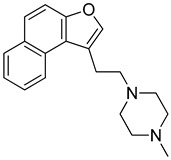	R_1_/R_2_	294.39
**R56**	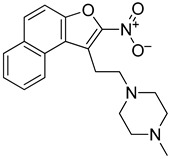	R_2_	339.39
**R62**	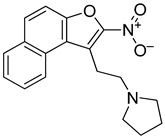	R_2_	310.13
**R75**	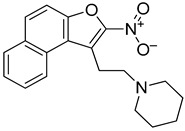	R_2_	324.37
**R76**	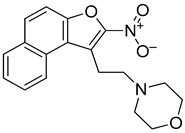	R_2_	326.13
**R118**	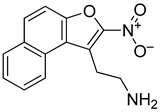	R_2_	256.08
**R158**	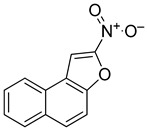	R_3_	213.04
**R114**	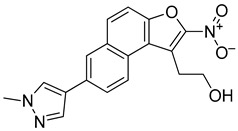	R_4_	337.11

## Data Availability

Data generated and analyzed in this current manuscript are available from the corresponding authors upon reasonable request.

## Supplementary Materials

The following supporting information can be downloaded at: https://www.mdpi.com/article/10.3390/genes13071144/s1, Materials and Methods: Detailed synthesis of each analog and Molecular modeling; Figure S1: Purity of NSC373981 by HPLC; Figure S2: Purity of NSC373981 synthesis (**R47**) and analogs by UPLC or HPLC; Figure S3: ^1^H NMR and ^13^C NMR spectra of synthesized NSC373981 (**R47**) and analogs; Figure S4: Replicate DNA polymerase stop gels of the *CARD11* G-quadruplex forming sequence; Figure S5: Unadjusted DNA polymerase stop gels of the *CARD11* wild-type and mutant G-quadruplex forming sequences for base identification; Figure S6: NSC373981 and **R158** stabilization of the *BCL2* G-quadruplex; Figure S7: Computational modeling of **R47** in an NMR generated structure of the *MYC* G-quadruplex; Figure S8: Cytotoxicity and determination of growth inhibitory concentrations of NSC373981 and analogs; Figure S9: Basal levels of *CARD11* mRNA; Figure S10: PDS qPCR of *CARD11*, *BCL2*, *MYC*, *KRAS*, and *TERT*; Figure S11: Replicate DNA polymerase stop gels of PDS and NSC373981 stabilization of the *CARD11* G-quadruplex forming sequence; Figure S12: Replicate DNA polymerase stop gel of NSC373981 and analogs with the *CARD11* G-quadruplex forming sequence; Table S1: Oligonucleotide sequences used in this study; Table S2: STR analysis of the DLBCL cell lines.

## References

[1] Alizadeh A.A., Eisen M.B., Davis R.E., Ma C., Lossos I.S., Rosenwald A., Boldrick J.C., Sabet H., Tran T., Yu X. (2000). Distinct types of diffuse large B-cell lymphoma identified by gene expression profiling. Nature.

[2] Lenz G., Wright G.W., Emre N.C., Kohlhammer H., Dave S.S., Davis R.E., Carty S., Lam L.T., Shaffer A.L., Xiao W. (2008). Molecular subtypes of diffuse large B-cell lymphoma arise by distinct genetic pathways. Proc. Natl. Acad. Sci. USA.

[3] Lohr J.G., Stojanov P., Lawrence M.S., Auclair D., Chapuy B., Sougnez C., Cruz-Gordillo P., Knoechel B., Asmann Y.W., Slager S.L. (2012). Discovery and prioritization of somatic mutations in diffuse large B-cell lymphoma (DLBCL) by whole-exome sequencing. Proc. Natl. Acad. Sci. USA.

[4] Morin R.D., Mungall K., Pleasance E., Mungall A.J., Goya R., Huff R.D., Scott D.W., Ding J., Roth A., Chiu R. (2013). Mutational and structural analysis of diffuse large B-cell lymphoma using whole-genome sequencing. Blood.

[5] Scott D.W., Wright G.W., Williams P.M., Lih C.J., Walsh W., Jaffe E.S., Rosenwald A., Campo E., Chan W.C., Connors J.M. (2014). Determining cell-of-origin subtypes of diffuse large B-cell lymphoma using gene expression in formalin-fixed paraffin-embedded tissue. Blood.

[6] Davis R.E., Ngo V.N., Lenz G., Tolar P., Young R.M., Romesser P.B., Kohlhammer H., Lamy L., Zhao H., Yang Y. (2010). Chronic active B-cell-receptor signalling in diffuse large B-cell lymphoma. Nature.

[7] Compagno M., Lim W.K., Grunn A., Nandula S.V., Brahmachary M., Shen Q., Bertoni F., Ponzoni M., Scandurra M., Califano A. (2009). Mutations of multiple genes cause deregulation of NF-kappaB in diffuse large B-cell lymphoma. Nature.

[8] Fowler N., Davis E. (2013). Targeting B-cell receptor signaling: Changing the paradigm. Hematol. Am. Soc. Hematol. Educ. Program..

[9] Ngo V.N., Young R.M., Schmitz R., Jhavar S., Xiao W., Lim K.H., Kohlhammer H., Xu W., Yang Y., Zhao H. (2011). Oncogenically active MYD88 mutations in human lymphoma. Nature.

[10] Chapuy B., Stewart C., Dunford A.J., Kim J., Kamburov A., Redd R.A., Lawrence M.S., Roemer M.G.M., Li A.J., Ziepert M. (2018). Molecular subtypes of diffuse large B cell lymphoma are associated with distinct pathogenic mechanisms and outcomes. Nat. Med..

[11] Schmitz R., Wright G.W., Huang D.W., Johnson C.A., Phelan J.D., Wang J.Q., Roulland S., Kasbekar M., Young R.M., Shaffer A.L. (2018). Genetics and Pathogenesis of Diffuse Large B-Cell Lymphoma. N. Engl. J. Med..

[12] Wright G.W., Huang D.W., Phelan J.D., Coulibaly Z.A., Roulland S., Young R.M., Wang J.Q., Schmitz R., Morin R.D., Tang J. (2020). A Probabilistic Classification Tool for Genetic Subtypes of Diffuse Large B Cell Lymphoma with Therapeutic Implications. Cancer Cell.

[13] Buchner M., Müschen M. (2014). Targeting the B-cell receptor signaling pathway in B lymphoid malignancies. Curr. Opin. Hematol..

[14] Woyach J.A., Furman R.R., Liu T.M., Ozer H.G., Zapatka M., Ruppert A.S., Xue L., Li D.H., Steggerda S.M., Versele M. (2014). Resistance mechanisms for the Bruton’s tyrosine kinase inhibitor ibrutinib. N. Engl. J. Med..

[15] Wilson W.H., Wright G.W., Huang D.W., Hodkinson B., Balasubramanian S., Fan Y., Vermeulen J., Shreeve M., Staudt L.M. (2021). Effect of ibrutinib with R-CHOP chemotherapy in genetic subtypes of DLBCL. Cancer Cell.

[16] Friedberg J.W., Sharman J., Sweetenham J., Johnston P.B., Vose J.M., Lacasce A., Schaefer-Cutillo J., De Vos S., Sinha R., Leonard J.P. (2010). Inhibition of Syk with fostamatinib disodium has significant clinical activity in non-Hodgkin lymphoma and chronic lymphocytic leukemia. Blood.

[17] Fontan L., Yang C., Kabaleeswaran V., Volpon L., Osborne M.J., Beltran E., Garcia M., Cerchietti L., Shaknovich R., Yang S.N. (2012). MALT1 small molecule inhibitors specifically suppress ABC-DLBCL in vitro and in vivo. Cancer Cell.

[18] Sun D., Hurley L.H. (2009). The importance of negative superhelicity in inducing the formation of G-quadruplex and i-motif structures in the c-Myc promoter: Implications for drug targeting and control of gene expression. J. Med. Chem..

[19] Bhattacharyya D., Mirihana Arachchilage G., Basu S. (2016). Metal Cations in G-Quadruplex Folding and Stability. Front. Chem..

[20] Huppert J.L., Balasubramanian S. (2007). G-quadruplexes in promoters throughout the human genome. Nucleic Acids Res..

[21] Brown R.V., Danford F.L., Gokhale V., Hurley L.H., Brooks T.A. (2011). Demonstration that drug-targeted down-regulation of MYC in non-Hodgkins lymphoma is directly mediated through the promoter G-quadruplex. J. Biol. Chem..

[22] Kendrick S., Muranyi A., Gokhale V., Hurley L.H., Rimsza L.M. (2017). Simultaneous Drug Targeting of the Promoter MYC G-Quadruplex and BCL2 i-Motif in Diffuse Large B-Cell Lymphoma Delays Tumor Growth. J. Med. Chem..

[23] Phan A.T., Kuryavyi V., Gaw H.Y., Patel D.J. (2005). Small-molecule interaction with a five-guanine-tract G-quadruplex structure from the human MYC promoter. Nat. Chem. Biol..

[24] Seenisamy J., Bashyam S., Gokhale V., Vankayalapati H., Sun D., Siddiqui-Jain A., Streiner N., Shin-Ya K., White E., Wilson W.D. (2005). Design and synthesis of an expanded porphyrin that has selectivity for the c-MYC G-quadruplex structure. J. Am. Chem. Soc..

[25] Siddiqui-Jain A., Grand C.L., Bearss D.J., Hurley L.H. (2002). Direct evidence for a G-quadruplex in a promoter region and its targeting with a small molecule to repress c-MYC transcription. Proc. Natl. Acad. Sci. USA.

[26] Hänsel-Hertsch R., Di Antonio M., Balasubramanian S. (2017). DNA G-quadruplexes in the human genome: Detection, functions and therapeutic potential. Nat. Rev. Mol. Cell Biol..

[27] Carvalho J., Mergny J.L., Salgado G.F., Queiroz J.A., Cruz C. (2020). G-quadruplex, Friend or Foe: The Role of the G-quartet in Anticancer Strategies. Trends Mol. Med..

[28] Kendrick S., Hurley L.H. (2010). The role of G-quadruplex/i-motif secondary structures as cis-acting regulatory elements. Pure Appl. Chem..

[29] Brooks T.A., Kendrick S., Hurley L. (2010). Making sense of G-quadruplex and i-motif functions in oncogene promoters. FEBS J..

[30] Bochman M.L., Paeschke K., Zakian V.A. (2012). DNA secondary structures: Stability and function of G-quadruplex structures. Nat. Rev. Genet..

[31] Kaiser C.E., Van Ert N.A., Agrawal P., Chawla R., Yang D., Hurley L.H. (2017). Insight into the Complexity of the i-Motif and G-Quadruplex DNA Structures Formed in the KRAS Promoter and Subsequent Drug-Induced Gene Repression. J. Am. Chem. Soc..

[32] Kang H.J., Cui Y., Yin H., Scheid A., Hendricks W.P.D., Schmidt J., Sekulic A., Kong D., Trent J.M., Gokhale V. (2016). A Pharmacological Chaperone Molecule Induces Cancer Cell Death by Restoring Tertiary DNA Structures in Mutant hTERT Promoters. J. Am. Chem. Soc..

[33] Desjardins M., Arjunaraja S., Stinson J.R., Dorjbal B., Sundaresan J., Niemela J., Raffeld M., Matthews H.F., Wang A., Angelus P. (2018). A Unique Heterozygous. Front. Immunol..

[34] Knies N., Alankus B., Weilemann A., Tzankov A., Brunner K., Ruff T., Kremer M., Keller U.B., Lenz G., Ruland J. (2015). Lymphomagenic CARD11/BCL10/MALT1 signaling drives malignant B-cell proliferation via cooperative NF-κB and JNK activation. Proc. Natl. Acad. Sci. USA.

[35] Lenz G., Davis R.E., Ngo V.N., Lam L., George T.C., Wright G.W., Dave S.S., Zhao H., Xu W., Rosenwald A. (2008). Oncogenic CARD11 mutations in human diffuse large B cell lymphoma. Science.

[36] Ajana A., Bideau J.-P., Cotrait M., Buisson J.-P., Demerseman P., Einhorn J., Royer R. (1988). Molecular and electronic structures of some mutagenic nitronaphthofurans: Structure—activity relationships. Eur. J. Med. Chem..

[37] Dexheimer T.S., Sun D., Hurley L.H. (2006). Deconvoluting the structural and drug-recognition complexity of the G-quadruplex-forming region upstream of the bcl-2 P1 promoter. J. Am. Chem. Soc..

[38] Brown R.V., Wang T., Chappeta V.R., Wu G., Onel B., Chawla R., Quijada H., Camp S.M., Chiang E.T., Lassiter Q.R. (2017). The Consequences of Overlapping G-Quadruplexes and i-Motifs in the Platelet-Derived Growth Factor Receptor β Core Promoter Nuclease Hypersensitive Element Can Explain the Unexpected Effects of Mutations and Provide Opportunities for Selective Targeting of Both Structures by Small Molecules To Downregulate Gene Expression. J. Am. Chem. Soc..

[39] De Cian A., Guittat L., Kaiser M., Saccà B., Amrane S., Bourdoncle A., Alberti P., Teulade-Fichou M.P., Lacroix L., Mergny J.L. (2007). Fluorescence-based melting assays for studying quadruplex ligands. Methods.

[40] Fleming A.M., Zhu J., Ding Y., Burrows C.J. (2019). Location dependence of the transcriptional response of a potential G-quadruplex in gene promoters under oxidative stress. Nucleic Acids Res..

[41] Du Z., Zhao Y., Li N. (2008). Genome-wide analysis reveals regulatory role of G4 DNA in gene transcription. Genome Res..

[42] Kumar N., Patowary A., Sivasubbu S., Petersen M., Maiti S. (2008). Silencing c-MYC expression by targeting quadruplex in P1 promoter using locked nucleic acid trap. Biochemistry.

[43] Nguyen G.H., Tang W., Robles A.I., Beyer R.P., Gray L.T., Welsh J.A., Schetter A.J., Kumamoto K., Wang X.W., Hickson I.D. (2014). Regulation of gene expression by the BLM helicase correlates with the presence of G-quadruplex DNA motifs. Proc. Natl. Acad. Sci. USA.

[44] Hänsel-Hertsch R., Beraldi D., Lensing S.V., Marsico G., Zyner K., Parry A., Di Antonio M., Pike J., Kimura H., Narita M. (2016). G-quadruplex structures mark human regulatory chromatin. Nat. Genet..

[45] David A.P., Margarit E., Domizi P., Banchio C., Armas P., Calcaterra N.B. (2016). G-quadruplexes as novel cis-elements controlling transcription during embryonic development. Nucleic Acids Res..

[46] Agarwal T., Roy S., Kumar S., Chakraborty T.K., Maiti S. (2014). In the sense of transcription regulation by G-quadruplexes: Asymmetric effects in sense and antisense strands. Biochemistry.

[47] Dai J., Carver M., Hurley L.H., Yang D. (2011). Solution structure of a 2:1 quindoline-c-MYC G-quadruplex: Insights into G-quadruplex-interactive small molecule drug design. J. Am. Chem. Soc..

[48] Trott O., Olson A.J. (2010). AutoDock Vina: Improving the speed and accuracy of docking with a new scoring function, efficient optimization, and multithreading. J. Comput. Chem..

[49] Catz S.D., Johnson J.L. (2001). Transcriptional regulation of bcl-2 by nuclear factor kappa B and its significance in prostate cancer. Oncogene.

[50] Hoesel B., Schmid J.A. (2013). The complexity of NF-κB signaling in inflammation and cancer. Mol. Cancer.

[51] La Rosa F.A., Pierce J.W., Sonenshein G.E. (1994). Differential regulation of the c-myc oncogene promoter by the NF-kappa B rel family of transcription factors. Mol. Cell Biol..

[52] Palumbo S.L., Ebbinghaus S.W., Hurley L.H. (2009). Formation of a unique end-to-end stacked pair of G-quadruplexes in the hTERT core promoter with implications for inhibition of telomerase by G-quadruplex-interactive ligands. J. Am. Chem. Soc..

[53] Cogoi S., Paramasivam M., Filichev V., Géci I., Pedersen E.B., Xodo L.E. (2009). Identification of a new G-quadruplex motif in the KRAS promoter and design of pyrene-modified G4-decoys with antiproliferative activity in pancreatic cancer cells. J. Med. Chem..

[54] Morgan R.K., Batra H., Gaerig V.C., Hockings J., Brooks T.A. (2016). Identification and characterization of a new G-quadruplex forming region within the kRAS promoter as a transcriptional regulator. Biochim. Biophys. Acta.

[55] Armas P., David A., Calcaterra N.B. (2017). Transcriptional control by G-quadruplexes: In vivo roles and perspectives for specific intervention. Transcription.

[56] Lovly C.M., Shaw A.T. (2014). Molecular pathways: Resistance to kinase inhibitors and implications for therapeutic strategies. Clin. Cancer Res..

[57] Song T., Chai G., Liu Y., Yu X., Wang Z., Zhang Z. (2016). Bcl-2 phosphorylation confers resistance on chronic lymphocytic leukaemia cells to the BH3 mimetics ABT-737, ABT-263 and ABT-199 by impeding direct binding. Br. J. Pharmacol..

