# Supplementary material for: Targeting a Novel G-Quadruplex in the CARD11 Oncogene Promoter with Naptho(2,1-b)furan-1-ethanol,2-nitro- Requires the Nitro Group
Source: Genes (Basel). 2022 Jun 25;13(7):1144. doi: 10.3390/genes13071144 (PMC9321325; doi:10.3390/genes13071144)
Supplement: Supplementary file 1 [file genes-13-01144-s001.zip › Supplementary Materials.pdf]

Supplementary Materials

Targeting a novel G-quadruplex in the *CARD11* oncogene promoter with naptho(2,1-b)furan-1-ethanol,2-nitro- requires the nitro group

Kennith Swafford[1], Baku Acharya[2], Thomas Raney[1], Mason McCrury[1], Debasmita Saha[2], Yingzhi Xu[1], Brendan Frett[2]*, Samantha Kendrick[1]*

[1]Department of Biochemistry and Molecular Biology, University of Arkansas for Medical Sciences, Little Rock, AR, USA; [2]Department of Pharmaceutical Sciences, University of Arkansas for Medical Sciences, Little Rock, AR, USA;

*Corresponding authors: Samantha Kendrick; Biochemistry and Molecular Biology Department, University of Arkansas for Medical Sciences, 4301 W Markham, slot #516, Little Rock, AR 72205; skendrick@uams.edu; (501)-686-5823

Brendan Frett; Pharmaceutical Sciences Department, University of Arkansas for Medical Sciences, 4301 W Markham, slot #522, Little Rock, AR 72205; BAFrett@uams.edu; (501)-526-0893

Materials and Methods

Synthesis of Analogs
The detailed synthesis of each analog is described below.

Synthesis of 2-(2-nitronaphtho[2,1-b]furan-1-yl)ethan-1-ol (**R47**):

To a mixture of naphthol (**1**, 2.0 g, 12.5 mmol) and ethyl-4-chloroacetoacetate (**2**, 2.34g, 14.2 mmol) was added concentrated H_2SO_4 at 0 °C over 30 minutes. The reaction mixture was stirred at room temperature for 3 hours followed by quenching dropwise into ice water. The reaction was stirred for 2 hours and the precipitate was filtered. The powder (4-(chloromethyl)-2H-benzo[h]chromen-2-one, **3**) obtained was used for next step without purification. Compound **3** (1 g, 4.1 mmol) was heated with 2 M NaOH (48 mL) solution for 16 hours. The reaction mixture was cooled before adjusting the pH to 5-6. The reaction mixture was extracted with ethyl acetate and concentrated under vacuum to get 2-(naphtho[2,1-b]furan-1-yl)acetic acid (**4**). The naphthofuran acetic acid (**4**, 0.75g, 2.9 mmol) was dissolved in THF (15 mL). Lithium aluminium hydride (334 mg, 8.8 mmol) was added slowly at 0 °C. The reaction progression and completion was monitored by TLC. Once the reaction was complete, aqueous HCl was added to the reaction mixture to adjust the pH to 5-6 and the aqueous phase was extracted with DCM three times. Following the extraction, the organic layer was washed with brine and evaporated under vacuum. The crude product was purified via flash chromatography to afford 2-(naphtho[2,1-b]furan-1-yl)ethan-1-ol (**5**). Concentrated nitric acid (60 µL) was added to 100 mg of **5** in 1.5 mL DCM at 0 °C. The reaction mixture was stirred at 0 °C until the consumption of **5**. The reaction mixture was diluted with water and extracted with ethyl acetate (3X) followed by washing with brine (3X). The organic solvent was evaporated and the crude mixture purified via flash chromatography to obtain 2-(2-nitronaphtho[2,1-b]furan-1-yl)ethan-1-ol (**R47**).

Synthesis of nitro-naphthofuran ethyl amines (**R56, R62, R75, R76, R118**):

p-toluene sulfonyl chloride (1.1 eq) was added portion wise to a mixture of **R47** (1 eq), dimethyl aminopyridine (0.05 eq), and triethylamine (2.5 eq) in DCM at 0 °C. The reaction was stirred at room temperature until consumption of starting material. Ethyl acetate and saturated sodium bicarbonate were added to the reaction mixture. The aqueous phase was extracted with EtOAc. The organic layer was washed with brine and evaporated under vacuum. The crude solid was purified via flash chromatography to afford 2-(2-nitronaphtho[2,1-b]furan-1-yl)ethyl 4-methylbenzenesulfonate (**7**). Compound **7** (1 mmol) was dissolved in acetonitrile followed by addition of the respective amine (10 eq). The reaction mixture was heated to 60 °C until the disappearance of starting material. The solvent was then evaporated, dissolved in ethyl acetate followed by addition of saturated bicarbonate solution. After the extraction of aqueous phase with EtOAc, the organic layer was evaporated under vacuum and the resulting crude product was purified via flash chromatography. All the amine derivatives were prepared with the same procedure except for R118. To afford R118, the starting material **7** was dissolved in 7 M methanolic ammonia solution followed by heating to 50 °C.

Synthesis of halogenated naphthofuran ethanol (**R67** and **R68**):

Compound 5 (30 mg, 0.14 mmol) was dissolved in hexafluoroisopropanol and purged with N_2. In a separate vial, chlorine (R67) or iodine (R68) succinimide (1.3 eq) was purged with N_2 followed by addition of compound 5 in solution. The reaction mixture was heated to 50 °C for 3 hours. Following the completion of reaction, the solvent was evaporated under vacuum, adsorbed onto silica, and purified via flash chromatography.

Synthesis of methylapyrazole nitro naphthol ethanol (**R114**):

R47 (100 mg, 0.4 mmol) was dissolved in 1.5 mL acetic acid. Bromine (192 mg, 1.2 mmol) was added dropwise. The reaction mixture was heated to 40 °C and monitored by TLC. After the complete consumption of starting material, 10% $Na_2S_2O_3$ and EtOAc were added. Following the workup, the organic layer was evaporated, and the crude product purified via flash chromatography to obtain **8**. Compound 8 (25 mg, 0.07 mmol), 1-methyl-4-pyrazole boronic acid pinacol ester (18 mg, 0.08 mmol),

Na_2CO_3 (15 mg, 0.14 mmol) was dissolved in 4:1 DMF: water. The solvent was vigorously bubbled for 10 minutes with argon gas. Tetrakistriphenylphosphine palladium (4 mg, 0.0035mmol) was added and the reaction was heated to 90 °C. The crude mixture was extracted with saturated sodium bicarbonate solution and ethyl acetate. The organic layer was evaporated, and the product was purified via flash chromatography.

Synthesis of nitro-naphthofuran (**R158**):

A mixture of naphthaldehyde (**9,** 50 mg, 0.3 mmol) and bromonitromethane (84 mg, 0.6 mmol) was dissolved in acetone (1.2 mL). Potassium carbonate (83 mg, 0.6 mmol) was added to the reaction mixture. The reaction was stirred at room temperature until naphthaldehyde was consumed. Following the complete removal of starting material, the acetone was evaporated to afford compound **10**. Acetic anhydride was added, and the reaction mixture was heated to reflux to obtain **R118**. Afterwards, the reaction mixture was added to water and extracted with EtOAc. The organic layer was then removed and the reaction was purified via flash chromatography.

Molecular Modeling

Molecular modeling studies were performed to predict the affinity and binding pose of naphtho[2,1-b]furans to the G-quadruplex using **R47** as the prototypical ligand. The c-MYC G-quadruplex solution structure was obtained and the quindoline ligand was removed (PDB ID: 2L7V) [47]. Protein preparation and ligand preparation were performed using ChemDraw, Discovery Studio 2021, and MGL Tools. Docking studies were performed using AutoDock Vina [48]. A grid box was defined to include the known ligand binding region within the G-quadruplex. **R47** was docked into this grid box to identify the lowest energy binding pose, which was predicted to have a ΔG value of -6.3 kcal/mol. The lowest energy binding pose was then visualized and analyzed using Discovery Studio 2021.

References

47. Dai, J.; Carver, M.; Hurley, L.H.; Yang, D. Solution structure of a 2:1 quindoline-c-MYC G-quadruplex: insights into G-quadruplex-interactive small molecule drug design. *J Am Chem Soc* **2011**, *133*, 17673-17680, doi:10.1021/ja205646q.
48. Trott, O.; Olson, A.J. AutoDock Vina: improving the speed and accuracy of docking with a new scoring function, efficient optimization, and multithreading. *J Comput Chem* **2010**, *31*, 455-461, doi:10.1002/jcc.21334.

Data File C:\CHEM32\1\DATA\DS-IMPURITY-1-20-2021\DB-373981000011.D
Sample Name: 373981-2nd batch-I-ACN

Sample: 373981 - 2nd Batch

===

```
Acq. Operator   : Debasmita
Acq. Instrument : Agilent 1290A                 Location : P1-A-03
Injection Date  : 1/20/2021 4:35:58 PM
                                              Inj Volume : 10.000 µl
Acq. Method     : C:\CHEM32\1\METHODS\DB-IMPURITY-COL1-ECLIPS-XDB-1-20-2021.M
Last changed    : 1/20/2021 4:33:23 PM by DS
                  (modified after loading)
Analysis Method : C:\CHEM32\1\METHODS\ZAF-388A-COL1-ECLIPS-XDB-1-162020.M
Last changed    : 2/9/2021 2:40:44 PM by Debasmita
                  (modified after loading)
Sample Info     : 373981-ACN-2a

Additional Info : Peak(s) manually integrated
```

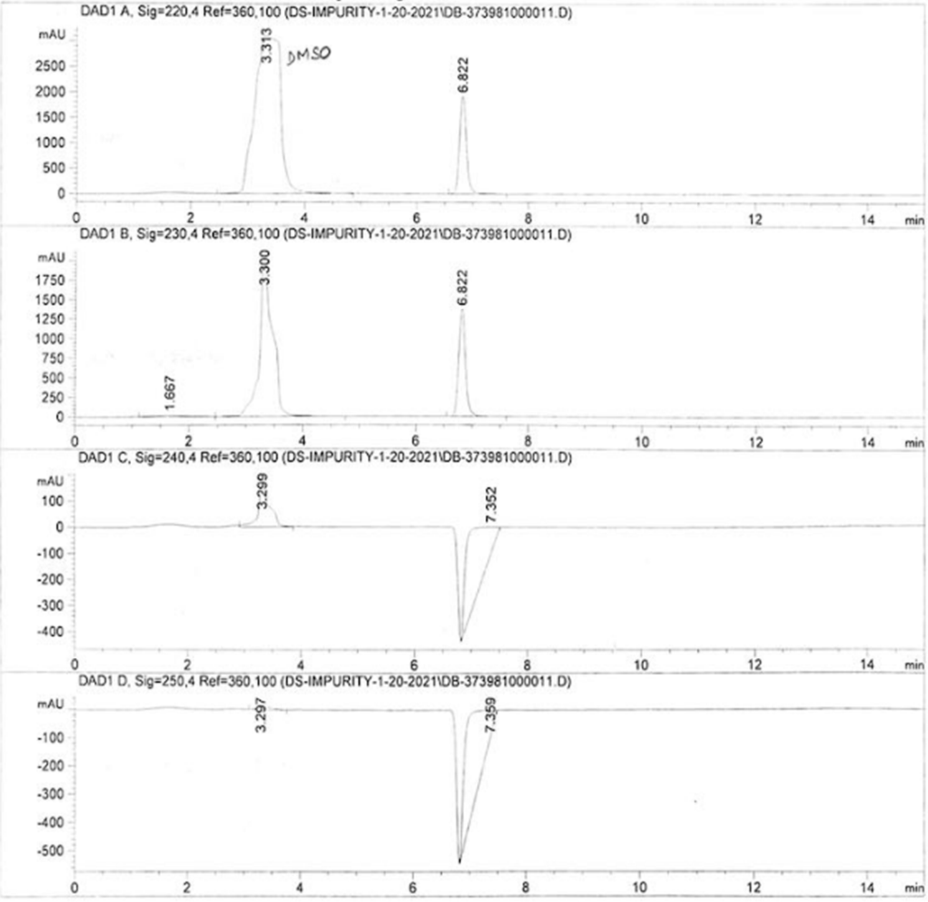

Supplementary Figure S1. Purity of NSC373981 by HPLC.

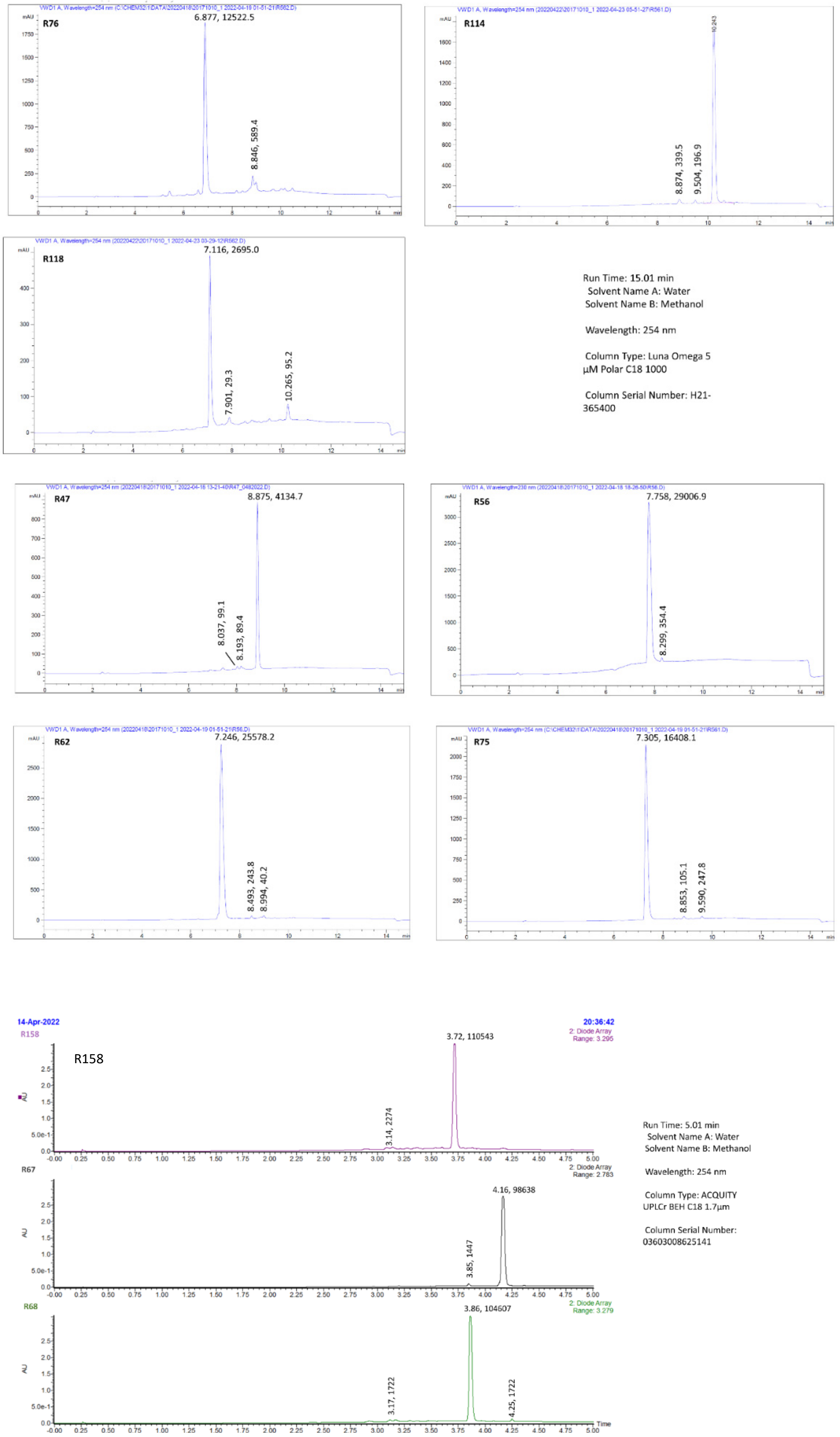

Supplementary Figure S2. Purity of NSC373981 synthesis (R47) and analogs by UPLC or HPLC.

R47:

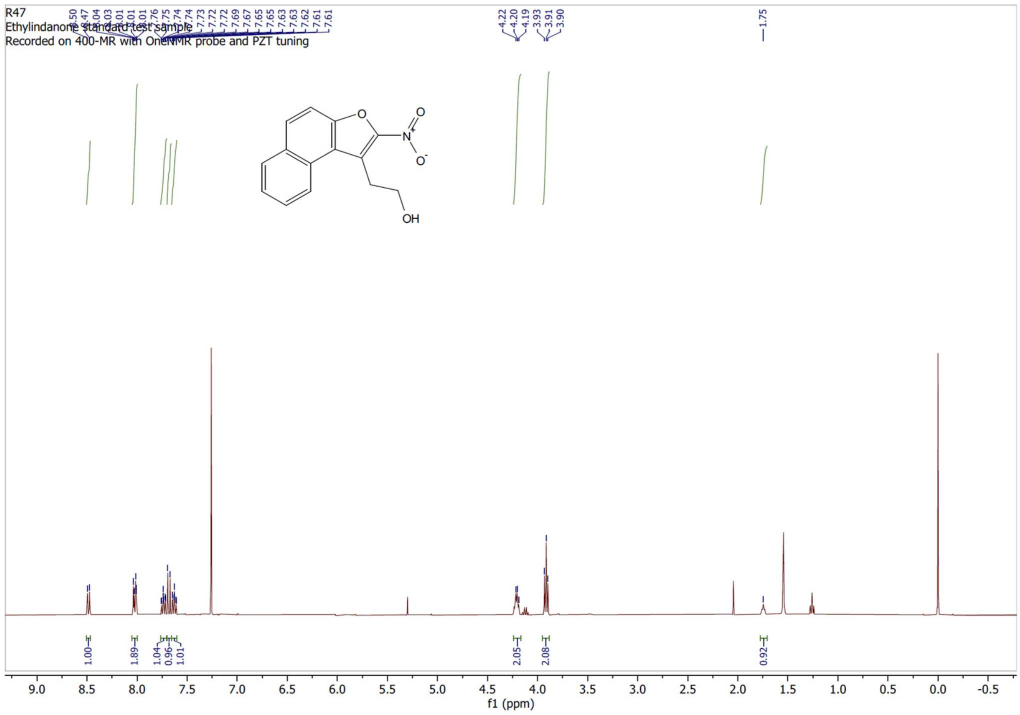
R47
Ethylindanone standard test sample
Recorded on 400-MR with OneNMR probe and PZT tuning
f1 (ppm)

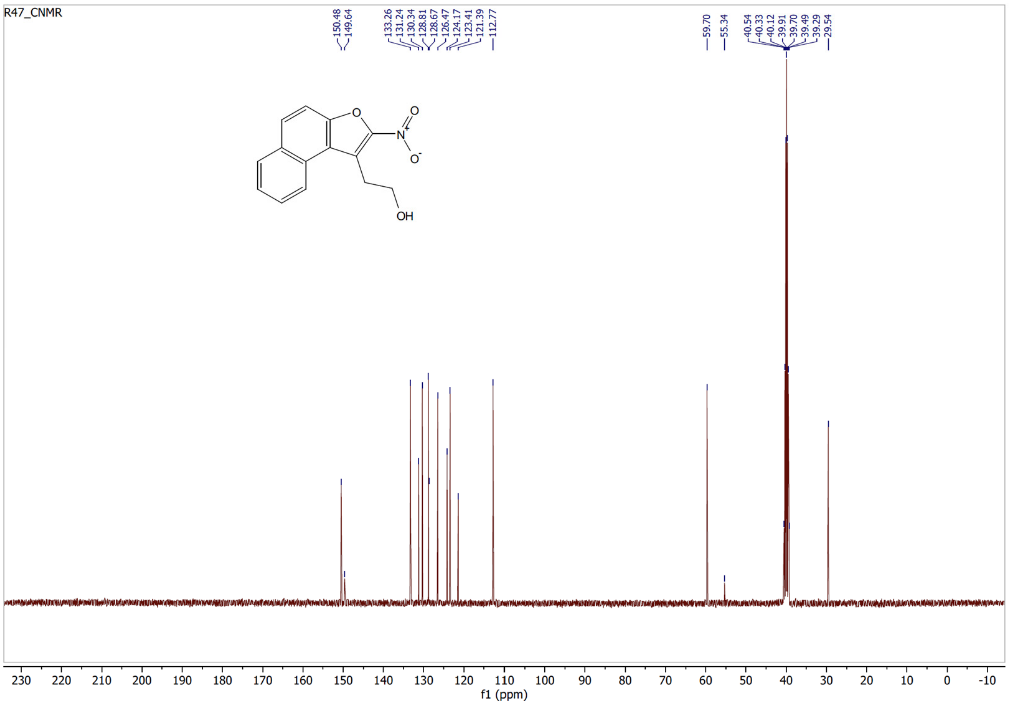
R47_CNMR
f1 (ppm)

R56:

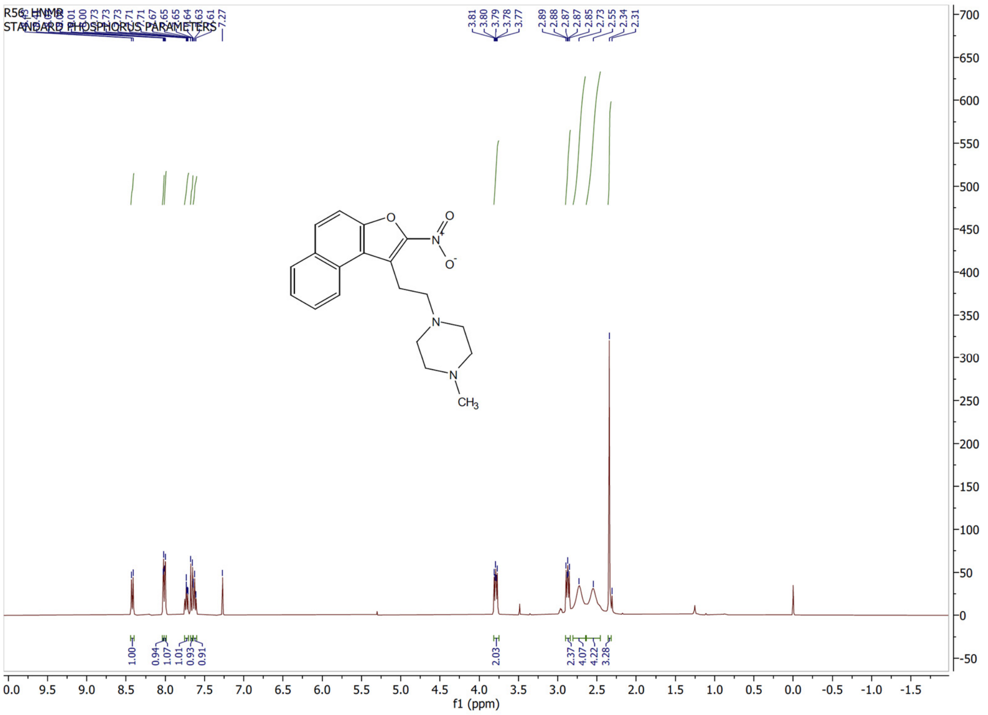

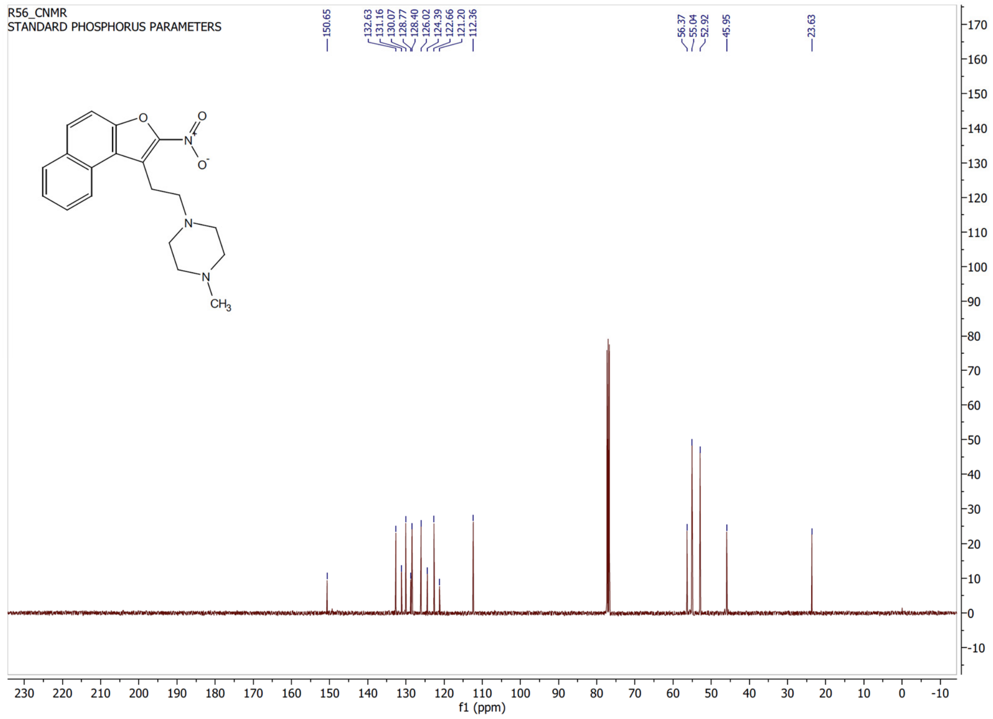

R67:

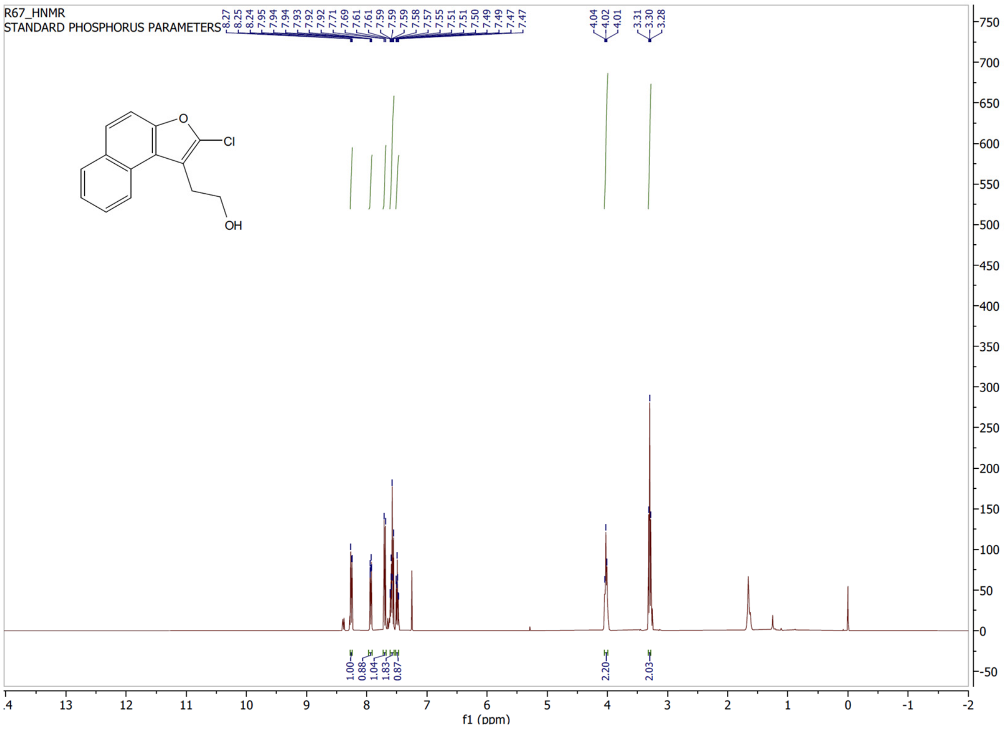

R67_HNMR
STANDARD PHOSPHORUS PARAMETERS
OH
Cl
O
f1 (ppm)

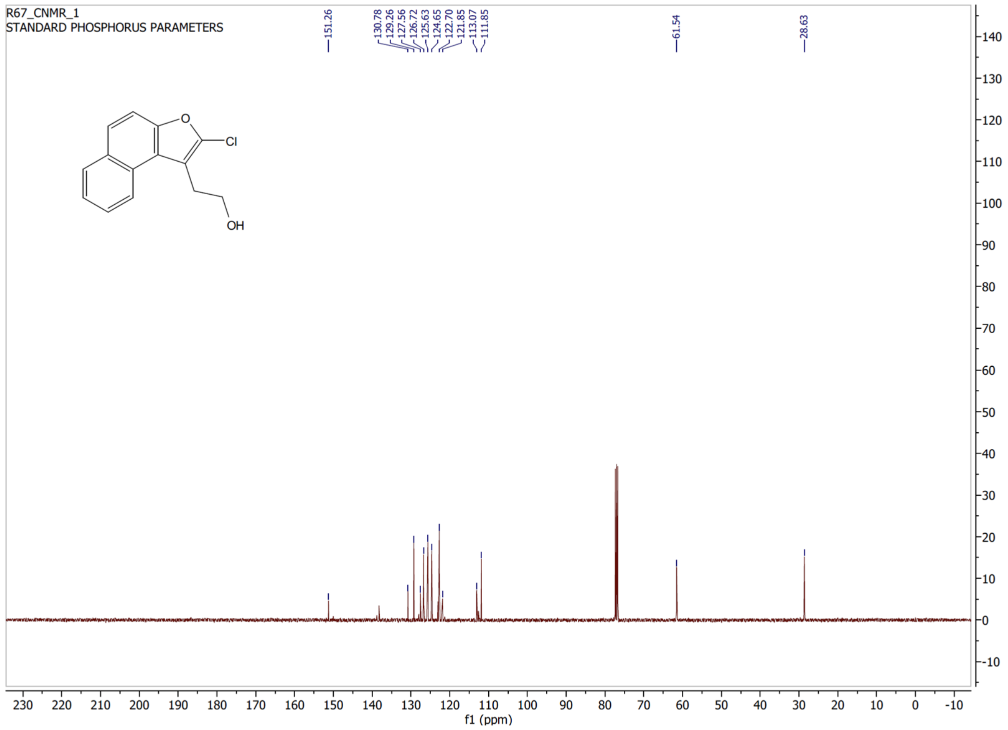

R67_CNMR_1
STANDARD PHOSPHORUS PARAMETERS
151.26
130.78
129.26
127.56
126.72
125.63
124.65
122.70
121.85
113.07
111.85
61.54
28.63
OH
Cl
O
f1 (ppm)

R68:

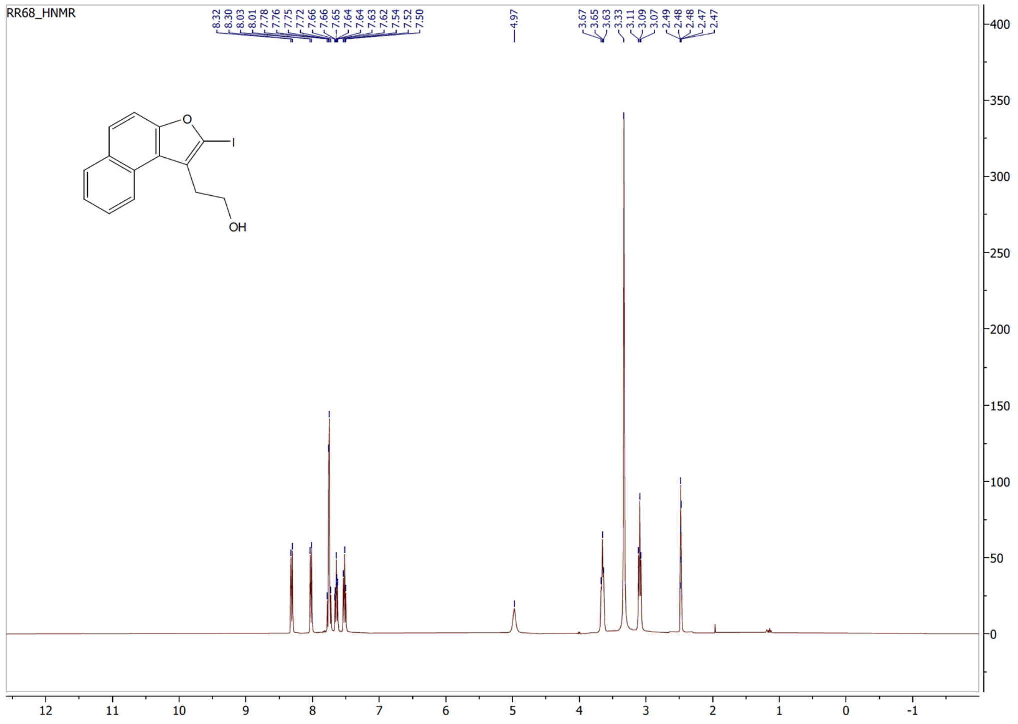

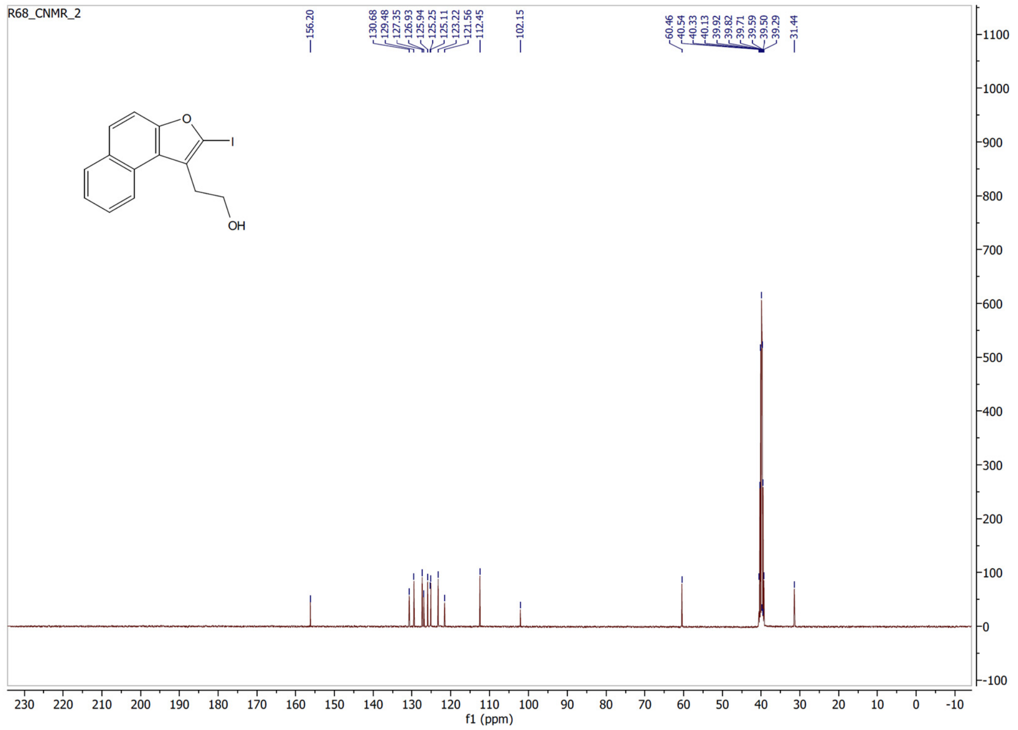

R75:

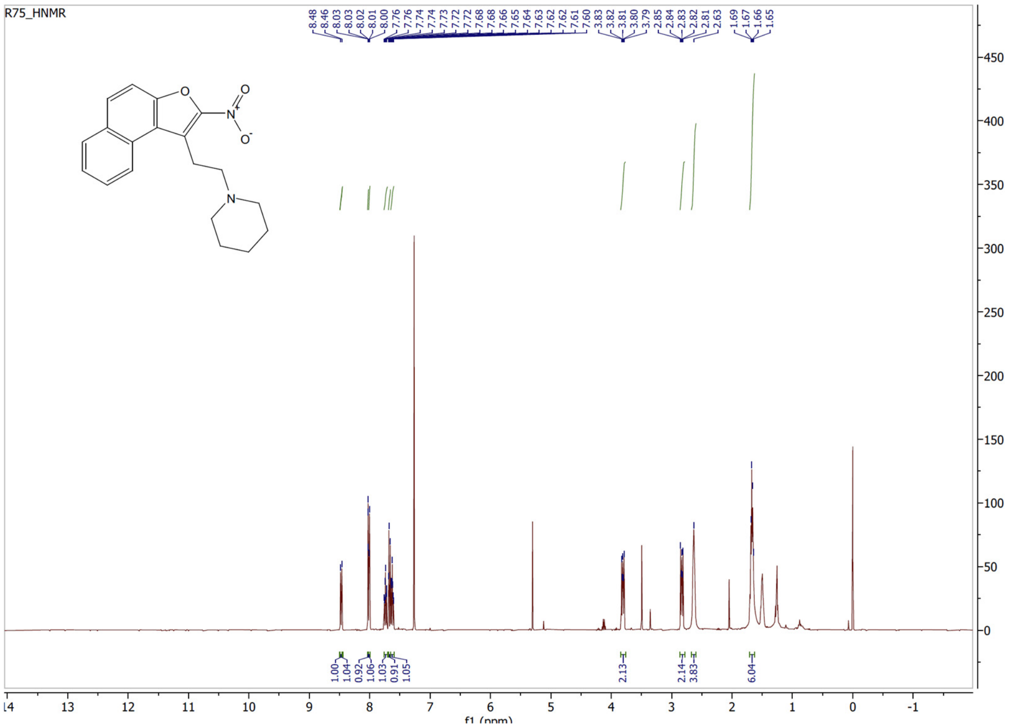
R75_HNMR
8.48 8.46 8.03 8.02 8.01 8.00 7.76 7.76 7.74 7.73 7.72 7.72 7.68 7.66 7.65 7.64 7.63 7.62 7.62 7.61 7.60 3.83 3.82 3.81 3.80 3.79 2.85 2.84 2.83 2.82 2.81 2.63 1.69 1.67 1.66 1.65
1.00 1.04 0.92 1.06 1.03 0.91 1.05 2.13 2.14 3.83 6.04

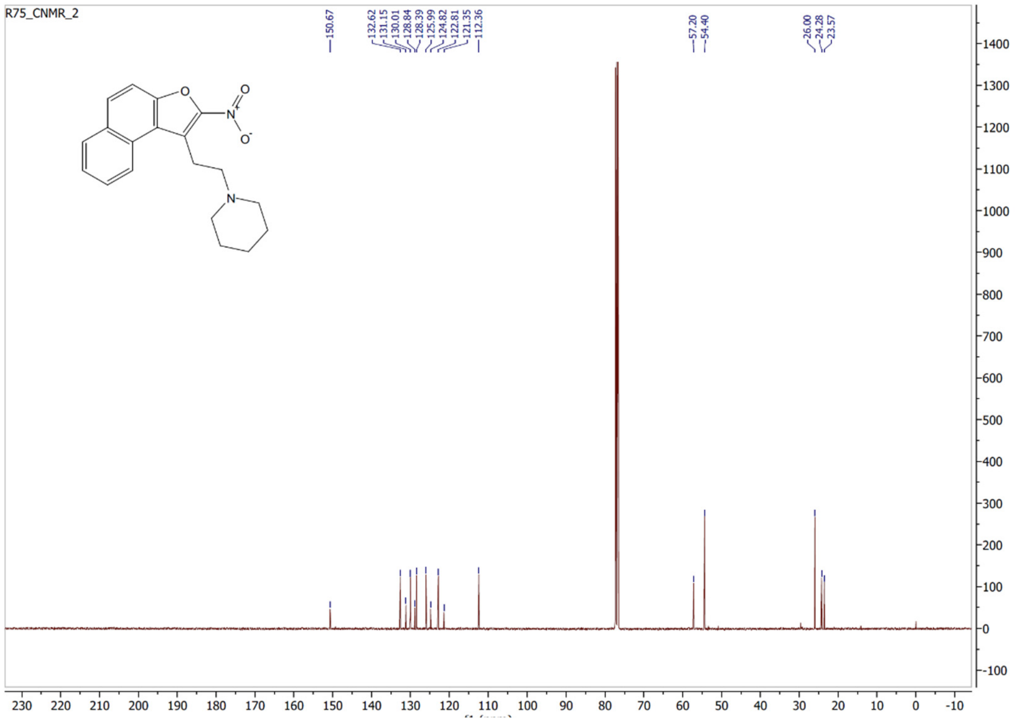
R75_CNMR_2
150.67 132.62 131.15 130.01 128.84 128.39 125.99 124.82 122.81 121.35 112.36 57.20 54.40 26.00 24.28 23.57

R76:

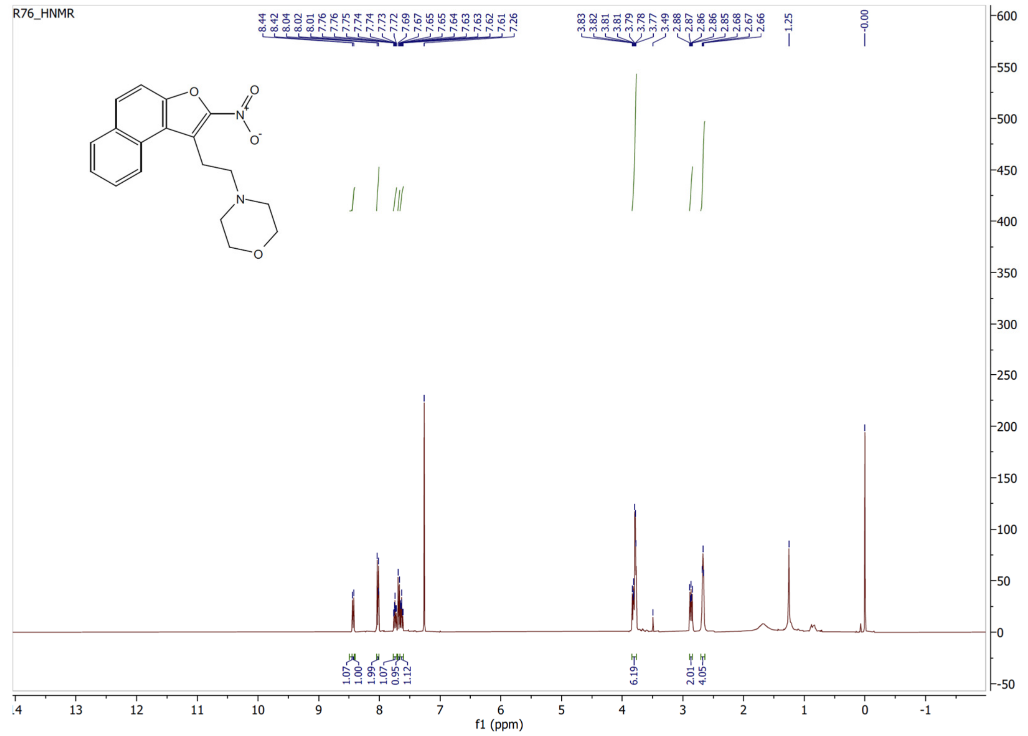

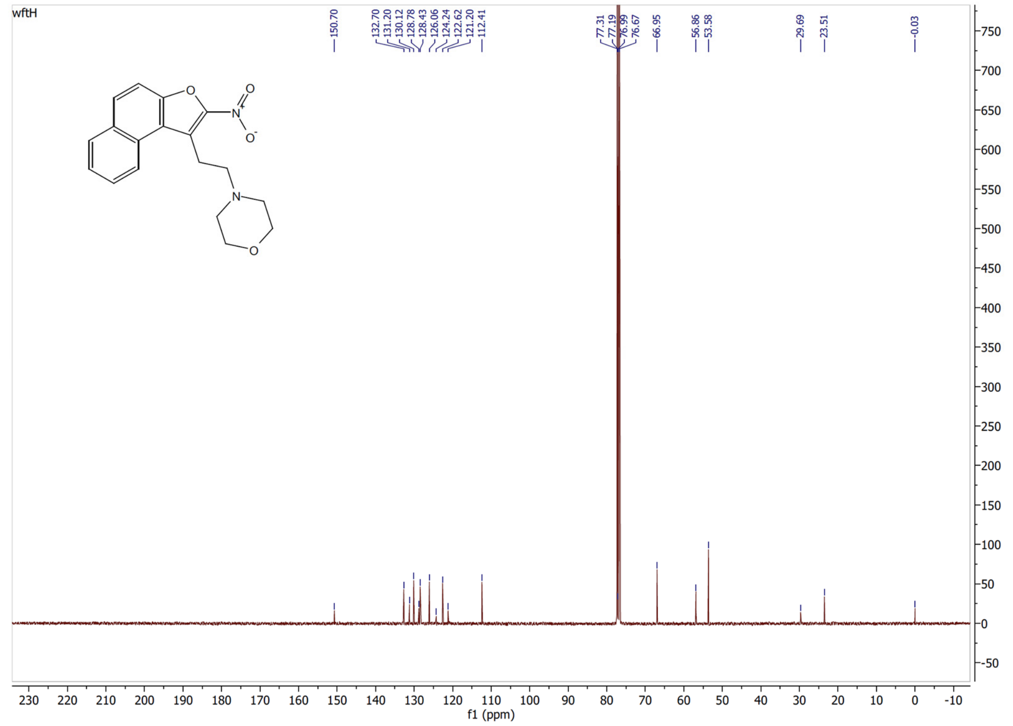

R62:

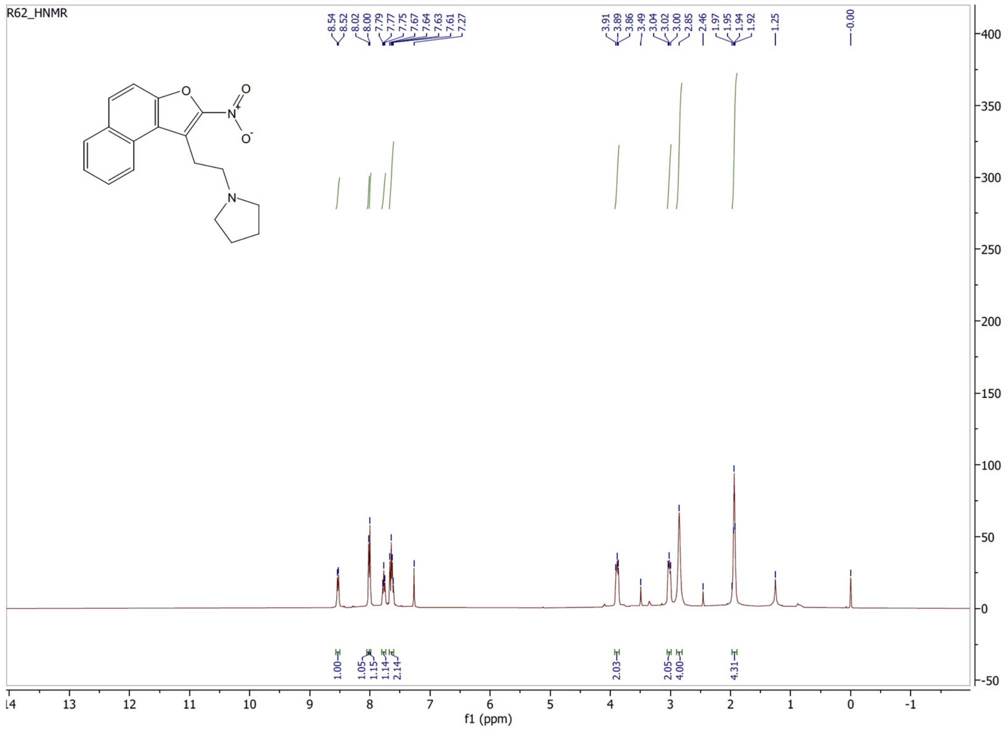
R62_HNMR
8.54
8.52
8.02
8.00
7.79
7.77
7.75
7.67
7.64
7.63
7.61
7.27
3.91
3.89
3.86
3.49
3.04
3.02
3.00
2.85
2.46
1.97
1.95
1.94
1.92
1.25
0.00
1.00
1.05
1.15
1.14
2.14
2.03
2.05
4.00
4.31
f1 (ppm)

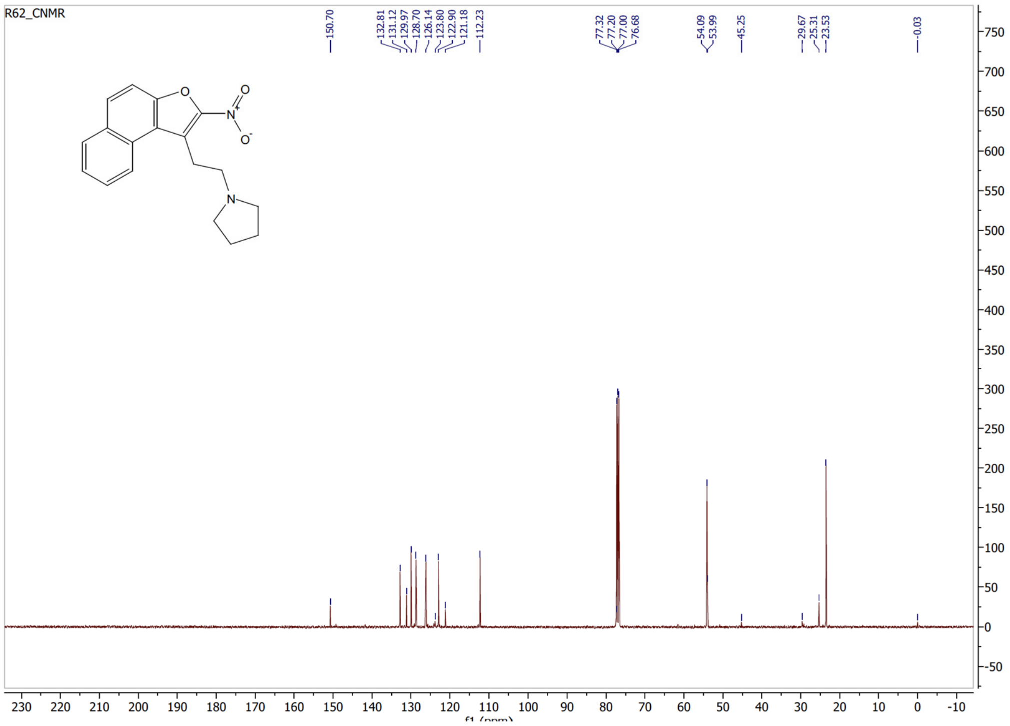
R62_CNMR
150.70
132.81
131.12
129.97
128.70
126.14
123.80
122.90
121.18
112.23
77.32
77.20
77.00
76.68
54.09
53.99
45.25
29.67
25.31
23.53
-0.03

R114:

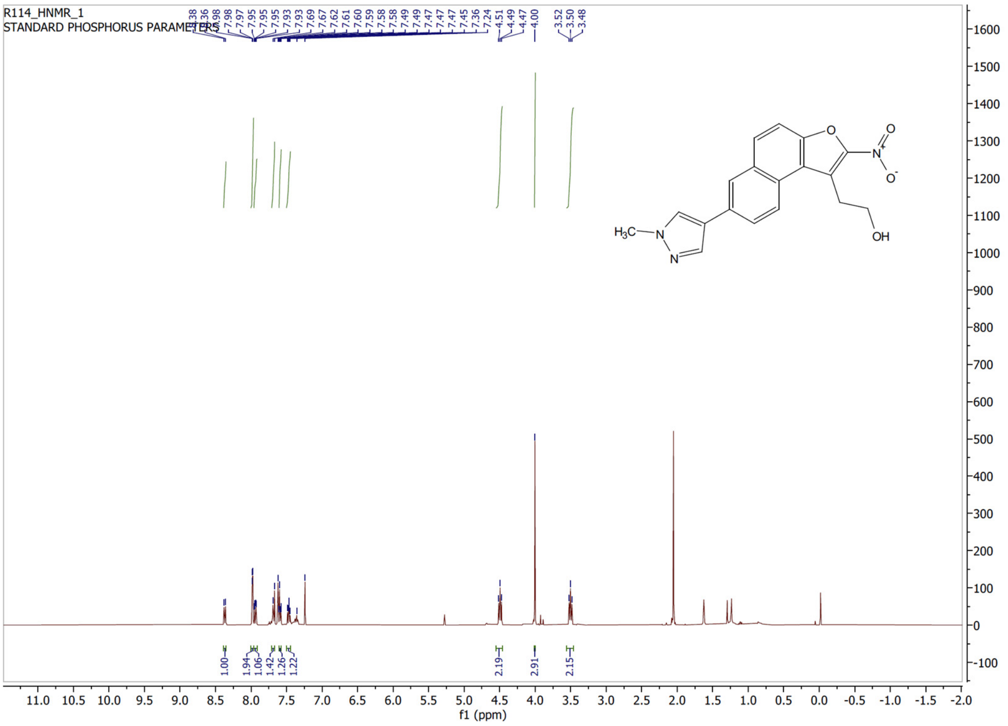
R114_HNMR_1
STANDARD PHOSPHORUS PARAMETERS
8.38
8.36
7.98
7.97
7.95
7.95
7.95
7.93
7.93
7.69
7.67
7.62
7.61
7.60
7.59
7.58
7.58
7.49
7.49
7.47
7.47
7.45
7.36
7.24
4.51
4.49
4.47
4.00
3.52
3.50
3.48
1.00
1.94
1.06
1.42
1.26
1.22
2.19
2.91
2.15
H_3C
N
N
O
N^+
O
O^-
OH
f1 (ppm)

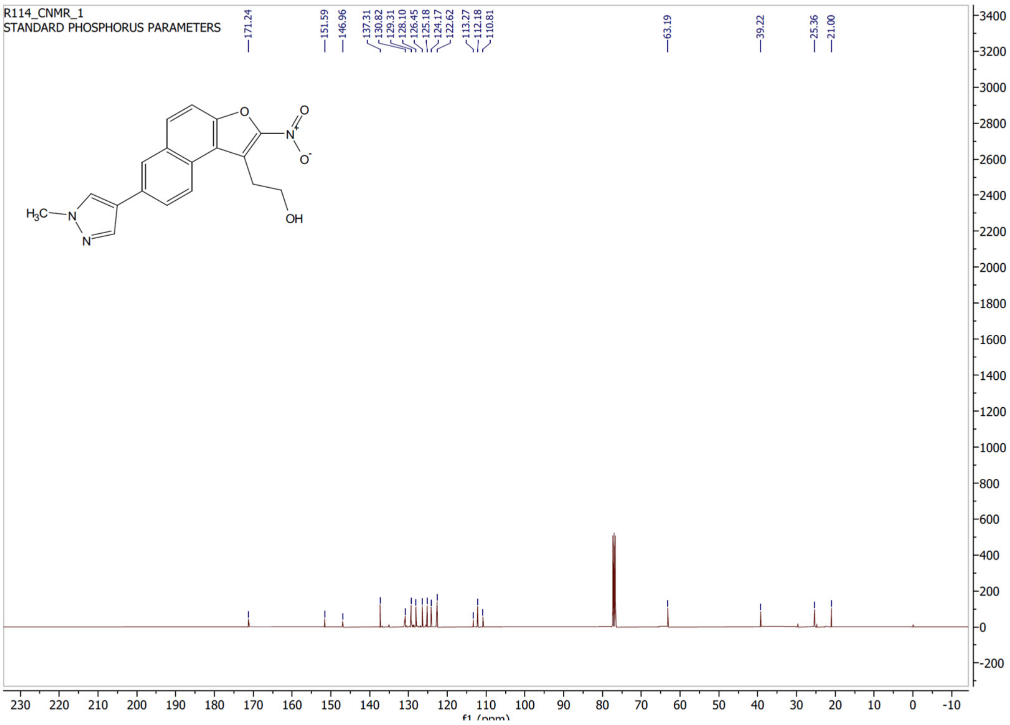
R114_CNMR_1
STANDARD PHOSPHORUS PARAMETERS
171.24
151.59
146.96
137.31
130.82
129.31
128.10
126.45
125.18
124.17
122.62
113.27
112.18
110.81
63.19
39.22
25.36
21.00
H_3C
N
N
O
N^+
O
O^-
OH
f1 (ppm)

R118:

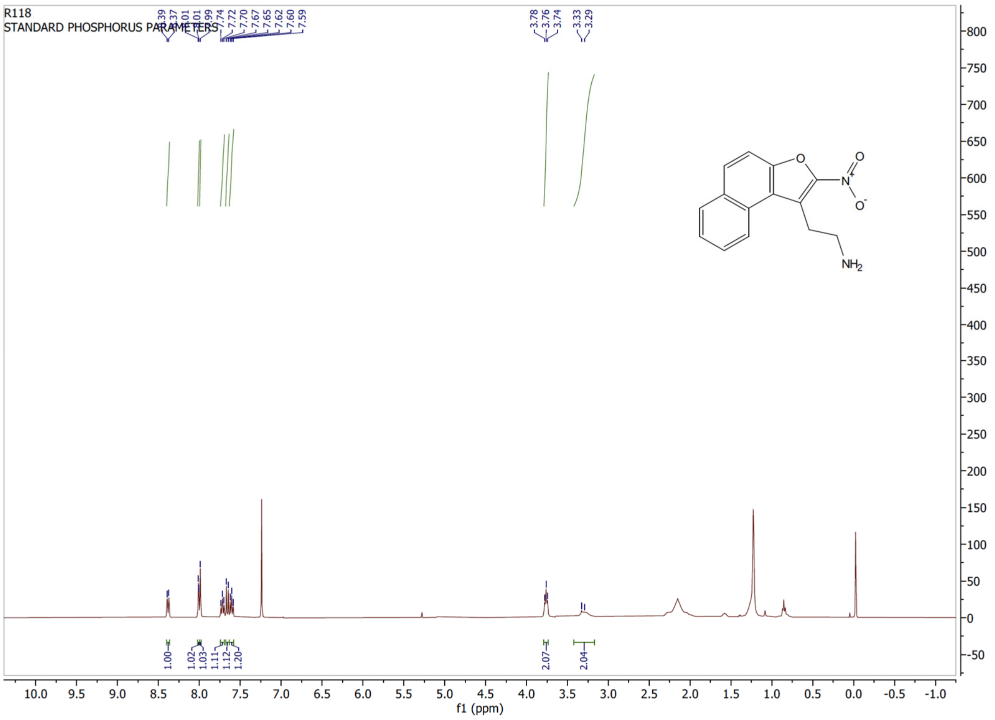
R118
STANDARD PHOSPHORUS PARAMETERS
8.39
8.37
8.01
8.01
7.99
7.74
7.72
7.70
7.67
7.65
7.62
7.60
7.59
3.78
3.76
3.74
3.33
3.29
1.00
1.02
1.03
1.11
1.12
1.20
2.07
2.04
f1 (ppm)
NH2

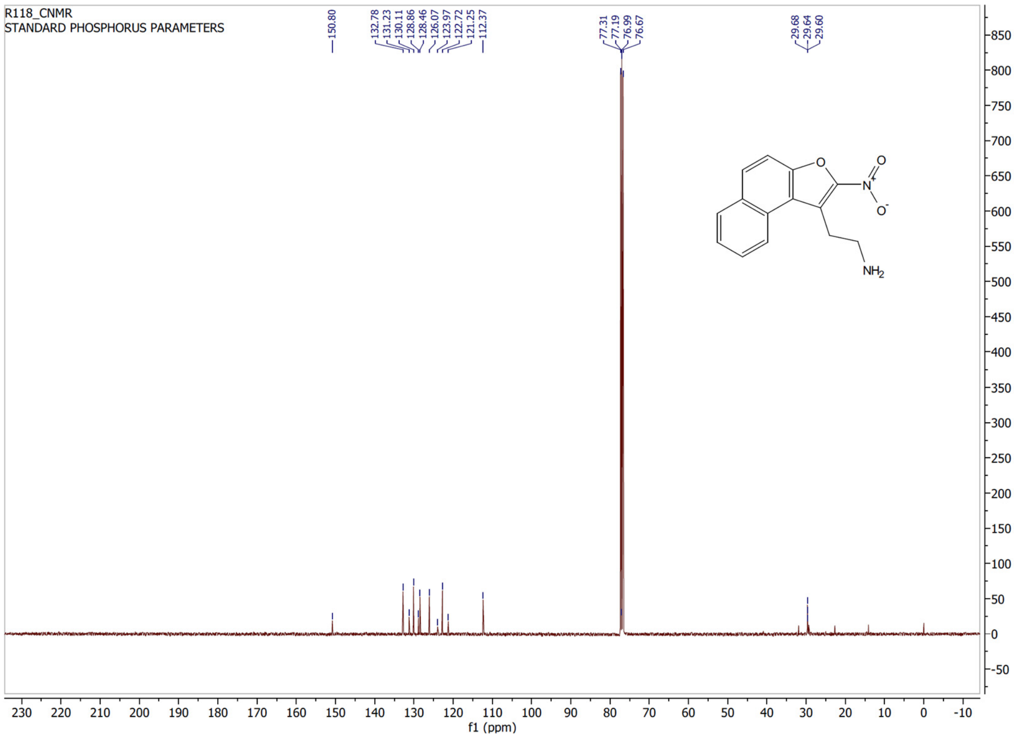
R118_CNMR
STANDARD PHOSPHORUS PARAMETERS
150.80
132.78
131.23
130.11
128.86
128.46
126.07
123.97
122.72
121.25
112.37
77.31
77.19
76.99
76.67
29.68
29.64
29.60
f1 (ppm)
NH2

R158:

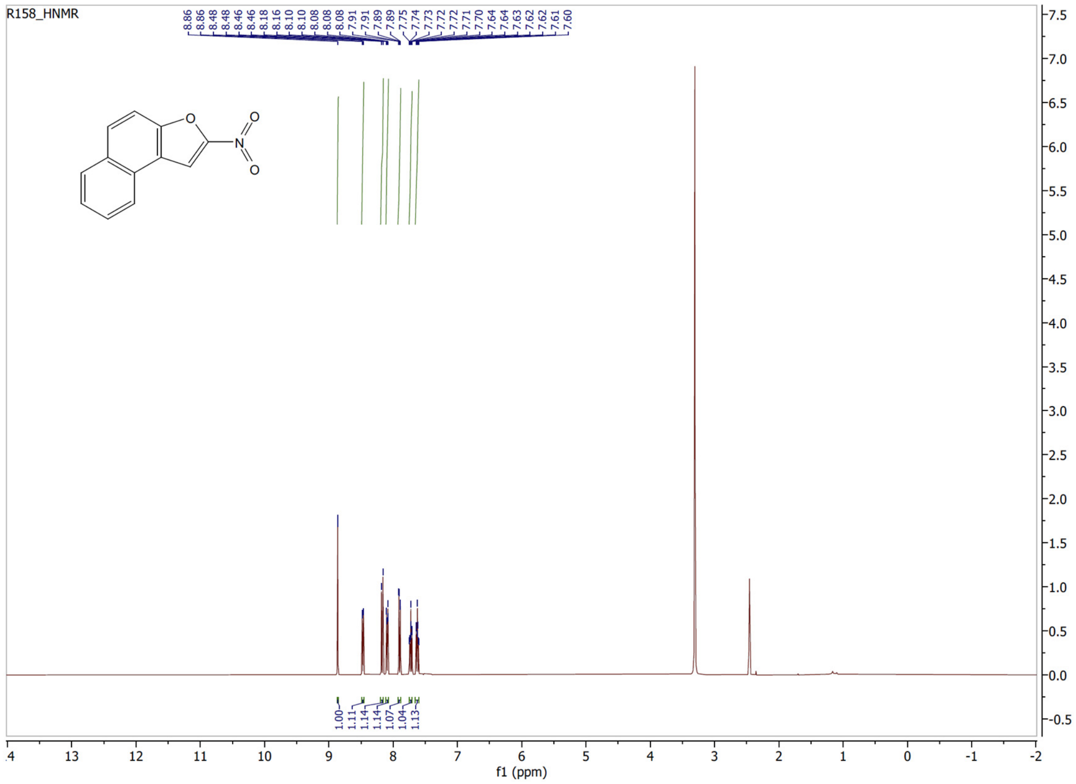

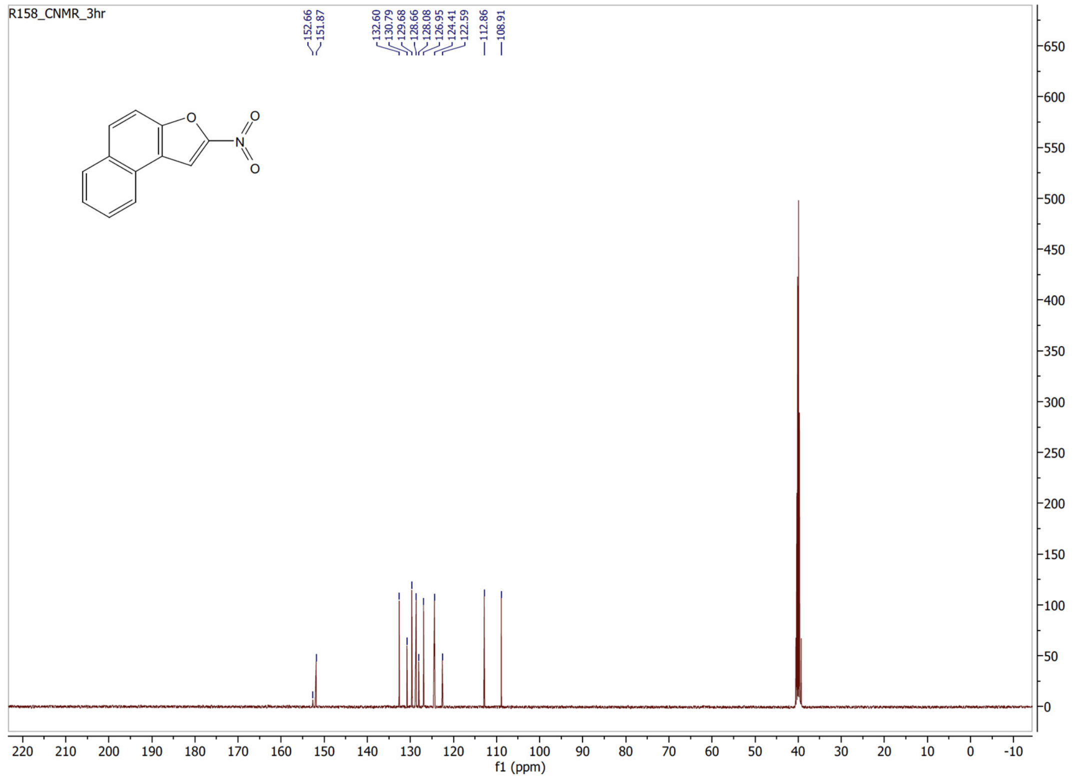

Supplementary Figure S3. ^{1}H NMR and ^{13}C NMR spectra of synthesized NSC373981 (R47) and analogs using an Agilent 400 MHz NMR spectrometer.

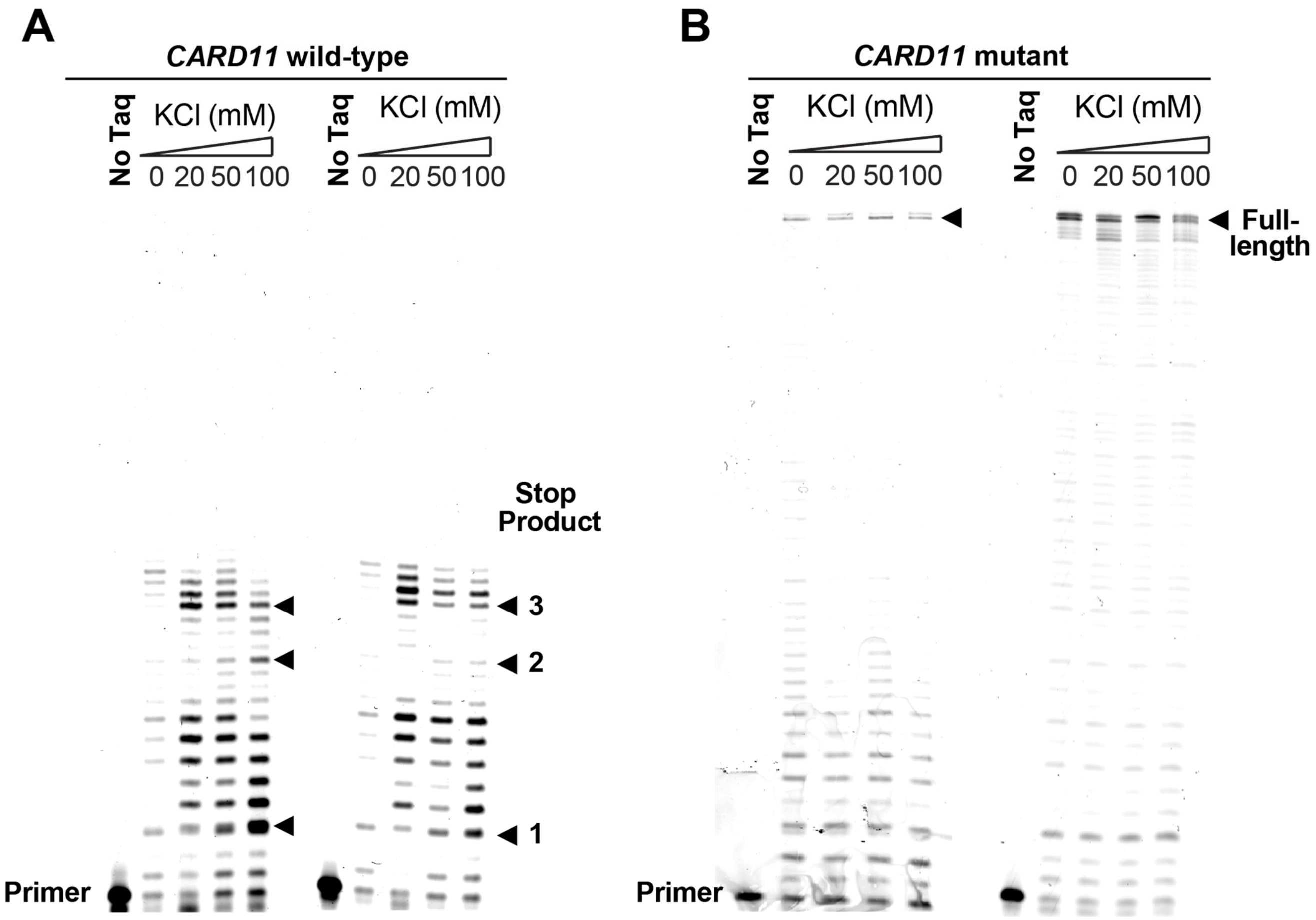

Supplementary Figure S4. Replicate DNA polymerase stop gels of the CARD11 G-quadruplex (G4) forming sequence. (**A**) and (**B**) Replicate DNA polymerase stop assay gels of two independent experiments of the Pu49 *CARD11* G4 wild-type and mutant sequences in the absence and presence of increasing concentrations of KCl.

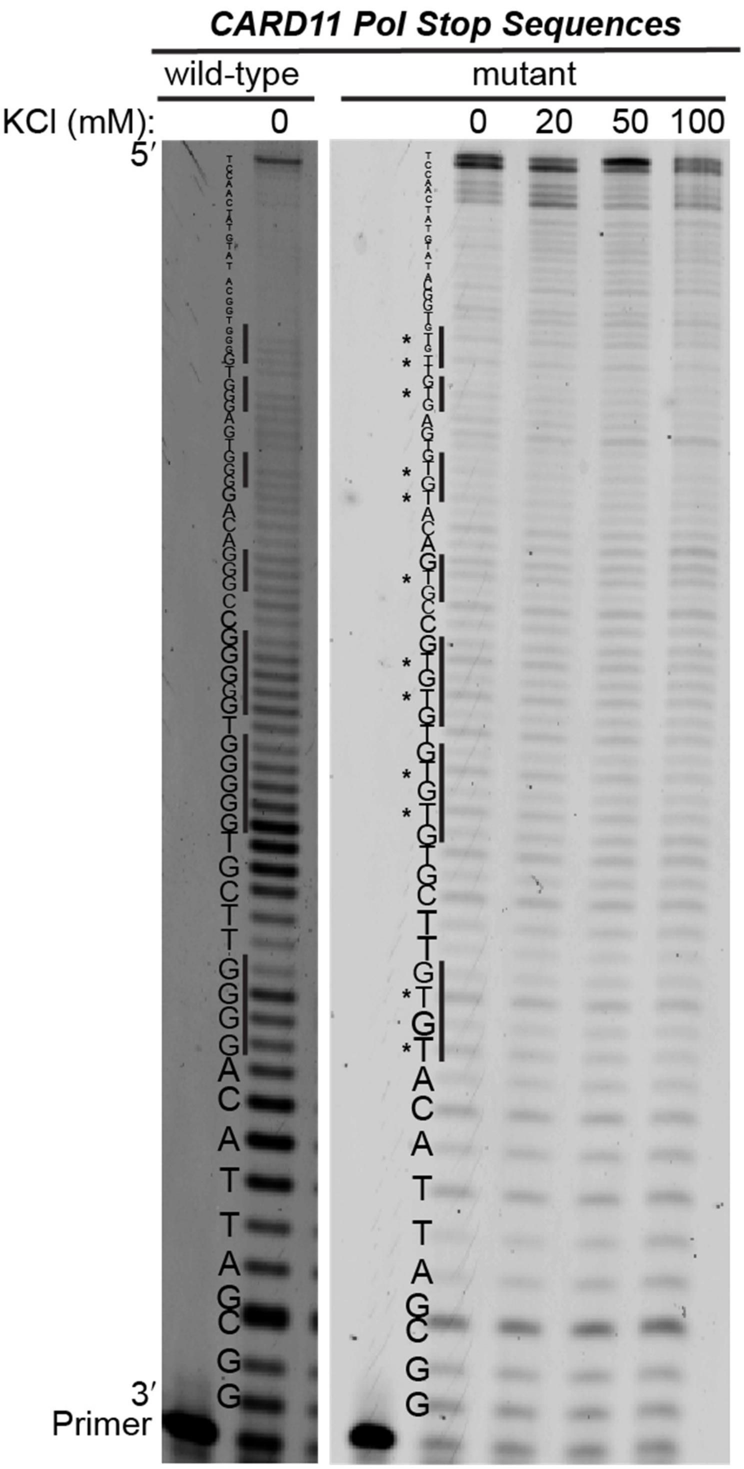

Supplementary Figure S5. Unadjusted DNA polymerase stop gels of the CARD11 G-quadruplex (G4) forming sequences. DNA polymerase stop assay gels of the *CARD11* G4 wild-type and mutant sequences in the absence or presence of increasing concentrations of KCl with the base identified to the corresponding band. Note, a 10-base and a 15-base extension was added to the 3′ and 5′ ends, respectively, of the Pu49 *CARD11* sequences (see Supplementary Table S1).

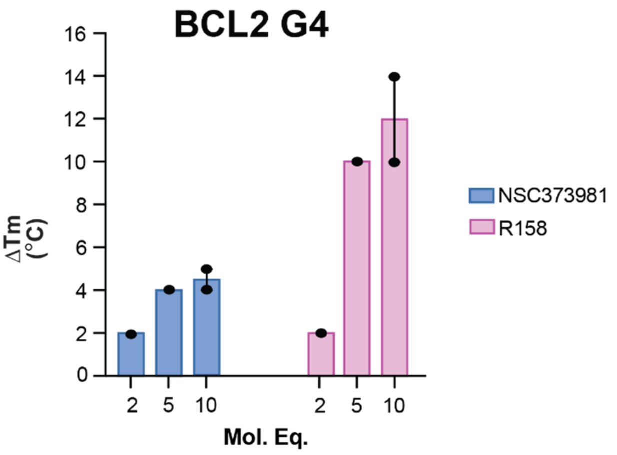

Supplementary Figure S6. NSC373981 and R158 stabilize the BCL2 G-quadruplex (G4). Difference in melting temperature (ΔT_m) of the BCL2 G4 from the FRET Melt assay performed at 20 mM KCl in the presence of increasing molar equivalents of NSC373981 (**blue**) or R158 (**pink**). ΔT_m are normalized to DMSO vehicle control.

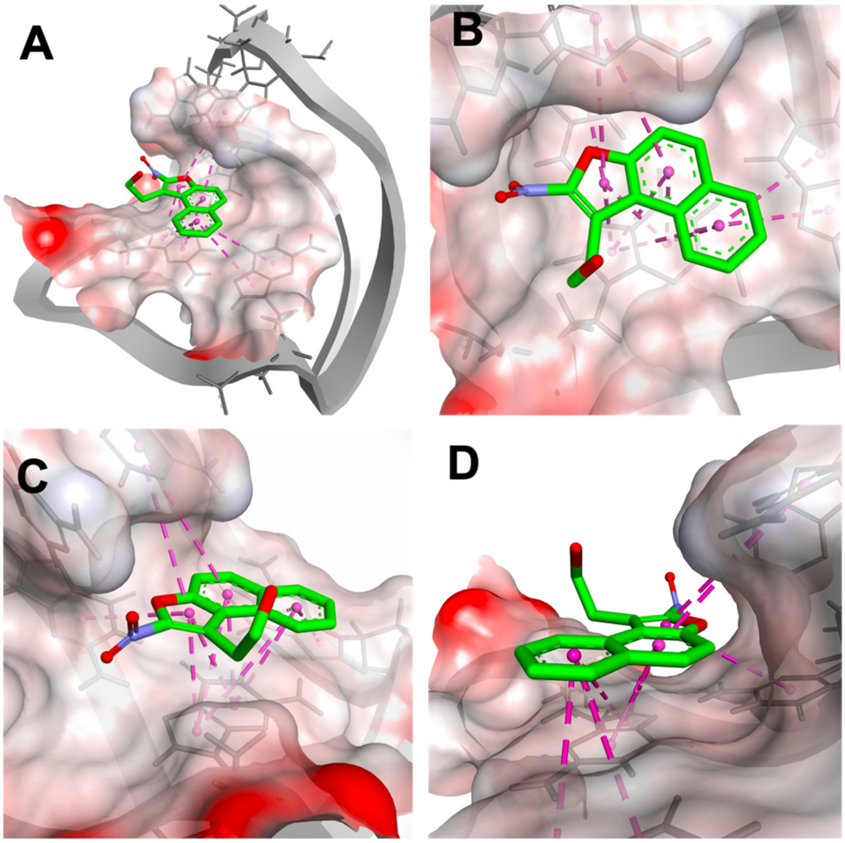

Supplementary Figure S7. Computational modeling of R47 (NSC373981) in an NMR generated structure of the MYC G-quadruplex (PBD: 2L7V). R47 binds to the G-quadruplex through Pi-Pi stacking interactions with guanine nucleotides as depicted in fuchsia, and the molecular surface is depicted as the interpolated surface charge of the structure (**A-D**). (**A**) Lowest energy confirmation of **R47** computationally modeled in the MYC G-quadruplex. The confirmation of **R47** is predicted to have an affinity of -6.3 kcal/mol for the complex. (**B**) The planar three-membered ring system of **R47** is electron rich and is predicted to interact with guanine nucleotides through a series of Pi-Pi stacking interactions. (**C**) The hydrophilic tail of **R47** is predicted to orient away from the G-quadruplex structure and into the solvent. (**D**) The electron rich ring system of **R47** is predicted to be held in place through interactions with multiple guanines that are both above and below the molecule. These guanines form an electron rich pocket that **R47** interacts with anchoring the molecule into the complex.

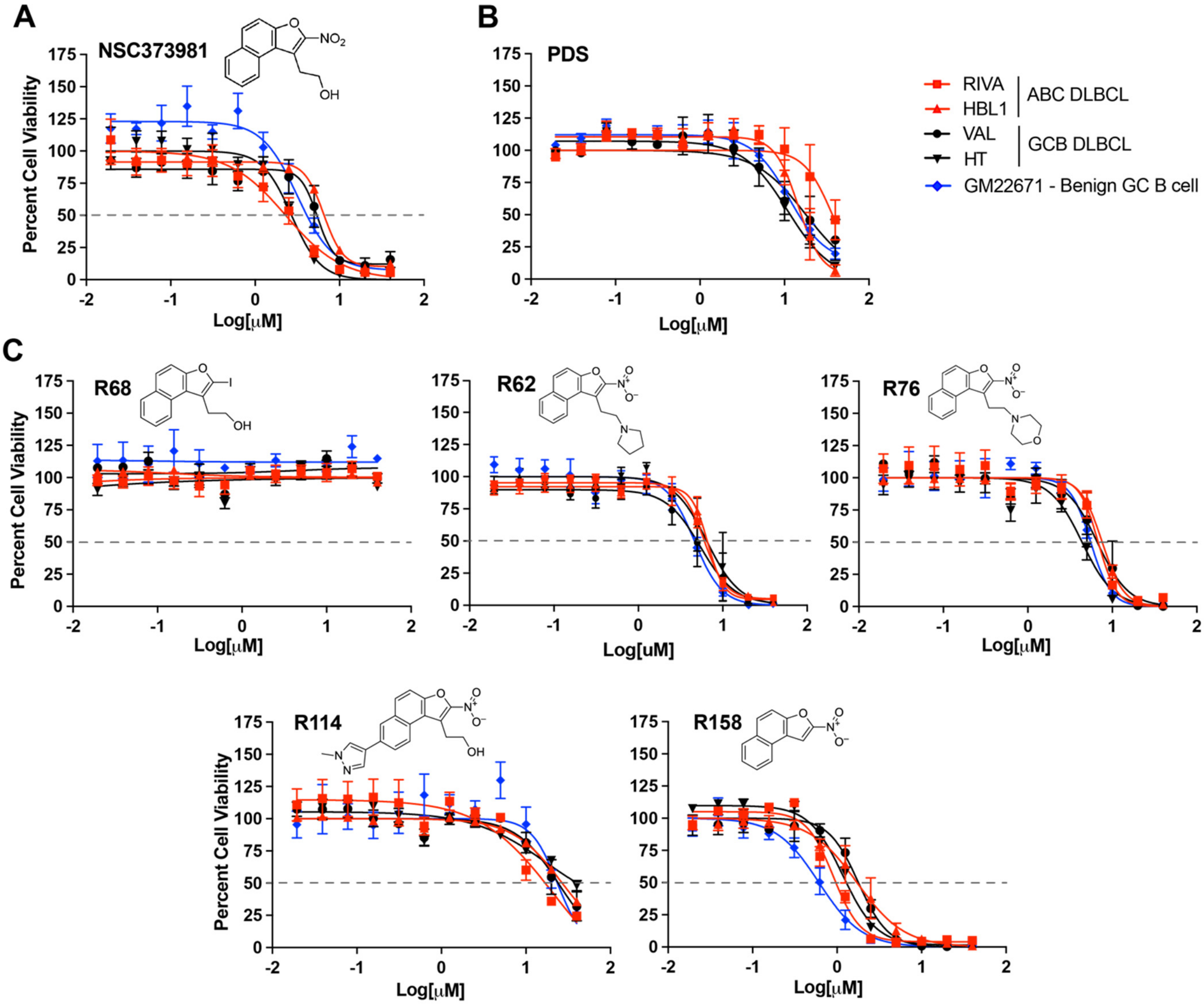

D

GI_{50} in μM (95% C.I.)

Compound	*ABC DLBCL* HBL1	*ABC DLBCL* RIVA	*GCB DLBCL* VAL	*GCB DLBCL* HT	*Benign GC B cells* GM22671
Parent					
NSC3733981	6.5 (5.7 - 6.5)	2.3 (1.7 - 3.1)	5.5 (4.1 - 7.2)	2.7 (2.3 - 3.2)	3.2 (2.3 - 4.3)
Analogs					
R68	> 40	> 40	> 40	> 40	> 40
R62	6.7 (5.7 - 7.6)	6.1 (5.3 - 7.0)	5.5 (3.8 - 9.1)	6.5 (5.0 - 8.4)	4.6 (3.9 - 5.5)
R76	7.6 (6.8 - 8.4)	6.8 (5.6 - 8.3)	6.6 (5.1 - 8.5)	4.4 (3.5 - 5.4)	5.5 (4.7 - 6.5)
R114	26 (25 - 29)	21 (8 - 78)	24 (19 - 31)	18 (7 - 62)	24 (17 - 34)
R158	1.7 (1.4 - 2.1)	0.9 (0.8 - 1.0)	1.8 (1.5 - 2.1)	1.2 (1.1 - 1.3)	0.6 (0.5 - 0.7)
PDS	14.6 (11.1 - 21.9)	36.9 (28.0 - 57.8)	18.0 (11.9 - 29.4)	10.9 (8.3 - 17.5)	11.4 (8.4 - 22.0)

Supplementary Figure S8. Cytotoxicity and determination of growth inhibitory concentrations of NSC373981 and analogs. (**A-C**) Percent viability of DLBCL and benign GC B cells as determined using the MTS cytotoxicity assay following treatment with NSC373981 (**A**), PDS (**B**), or analogs (**C**). Data represent mean ± SEM from three independent experiments, except for RIVA cells and PDS, which represent two independent experiments. (**C**) Growth inhibitory concentrations (GI_{50}) for each compound in μM with the 95% Confidence Interval (C.I.) calculated from two or three independent experiments. All compounds were incubated with each cell line for 72 h.

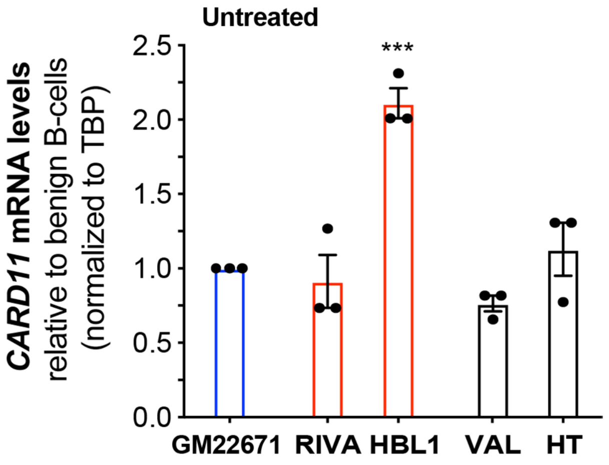

Supplementary Figure S9. Basal levels of *CARD11* mRNA. *CARD11* mRNA expression in ABC DLBCL cell lines, RIVA and HBL, (red) and GCB DLBCL cell lines, VAL and HT (black) compared to the benign GC B cells, GM22671 (blue) as detected by qPCR. Data represent mean ± SEM from three independent collections of untreated cells. ***adjusted *P*-value = 0.0003 using a one-way ANOVA with Dunnett's multiple test correction.

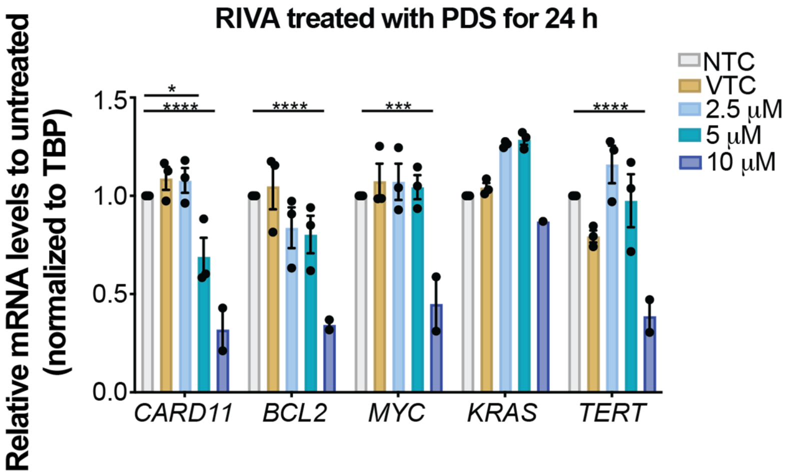

Supplementary Figure S10. PDS lowers *CARD11* mRNA. *CARD11* mRNA expression in the ABC DLBCL cell line, RIVA as detected by qPCR following 24 h of PDS treatment. No treatment control (NTC, media only) and DMSO vehicle treated control (VTC) were used for comparison. Data represent mean ± SEM from three independent experiments, except for 10 µM PDS treatments, which represent two independent experiments. ***adjusted *P*-value = 0.0003 using a one-way ANOVA with Dunnett's multiple test correction.

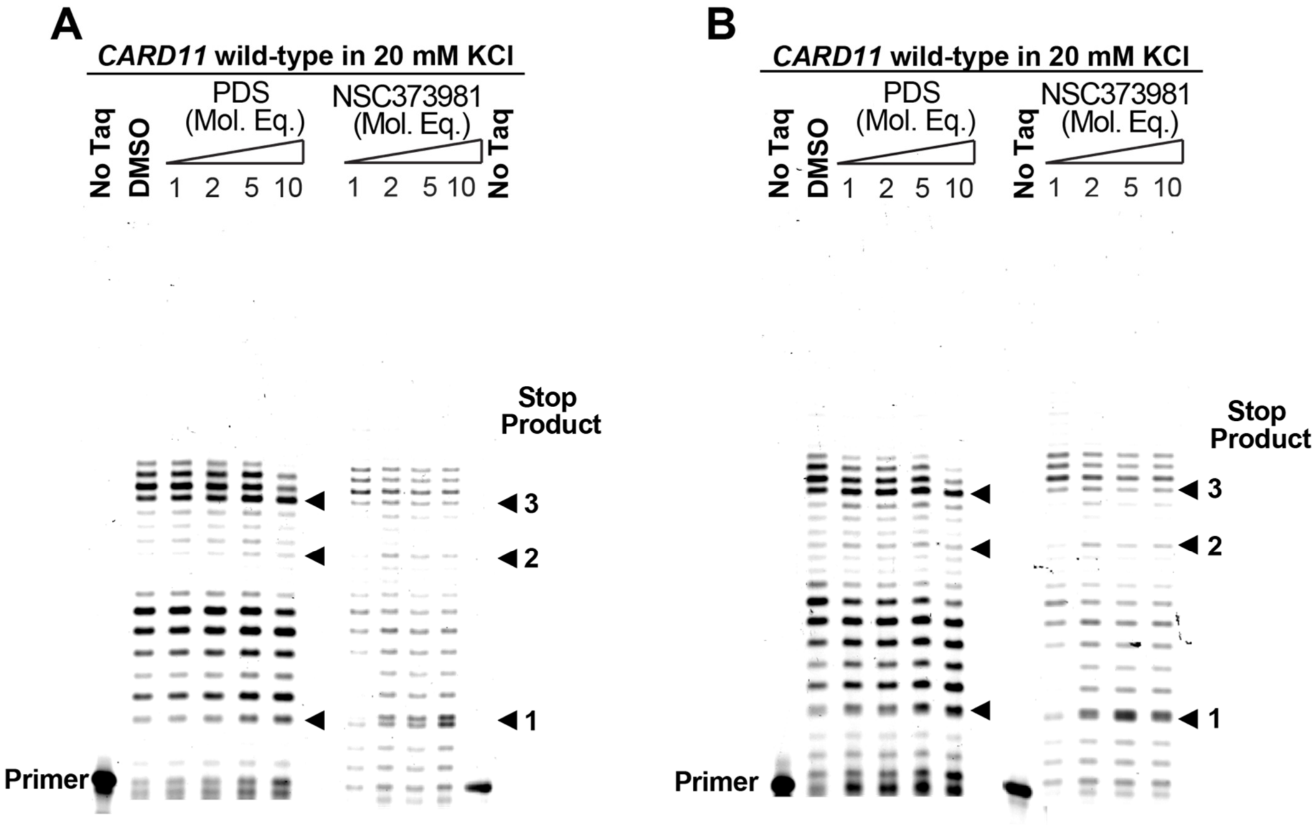

Supplementary Figure S11. Replicate DNA polymerase stop gels of PDS and NSC373981 stabilization of the CARD11 G-quadruplex (G4) forming sequence. (**A**) and (**B**) Replicate DNA polymerase stop assay gels of two independent experiments of the Pu49 *CARD11* G4 wild-type sequence in the absence and presence of increasing concentrations of KCl.

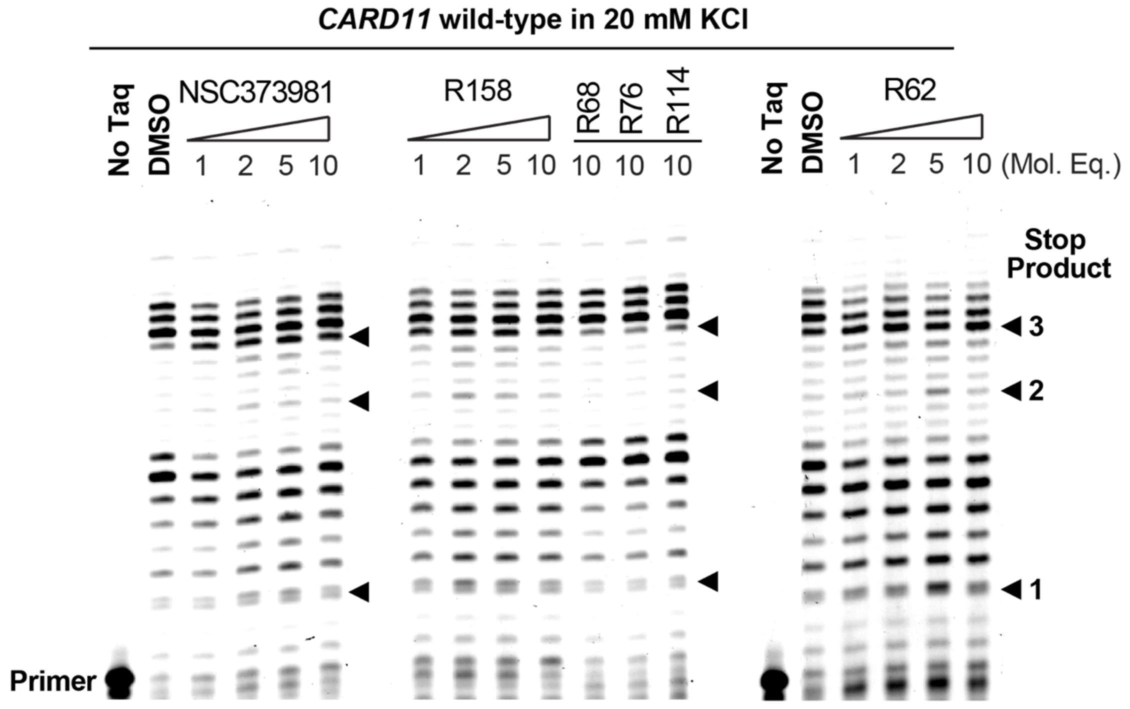

Supplementary Figure S12. Replicate DNA polymerase stop gels of NSC373981 analogs stabilization of the CARD11 G-quadruplex (G4) forming sequence. Replicate DNA polymerase stop assay gels of two independent experiments of the Pu49 *CARD11* G4 wild-type sequence in the absence and presence of increasing concentrations of KCl.
